# IgM Antibodies Targeting Malondialdehyde Promote Complement‐Mediated Liver Injury in Alcohol‐Related Liver Disease

**DOI:** 10.1111/liv.70356

**Published:** 2025-09-17

**Authors:** Dragana Rajcic, Beatriz Pereira da Silva, Taras Baranovskyi, Benedikt Simbrunner, Benedikt S. Hofer, Constanze Hoebinger, Tereza Duckova, Nikolina Papac‐Milicevic, Stephan Listabarth, Sabine Weber, Benjamin Vyssoki, Daniel König, Katharina Burger, Ina Bergheim, Christoph J. Binder, Mattias Mandorfer, Thomas Reiberger, Tim Hendrikx

**Affiliations:** ^1^ Department of Laboratory Medicine Medical University of Vienna Vienna Austria; ^2^ Division of Gastroenterology and Hepatology, Department of Medicine III Medical University of Vienna Vienna Austria; ^3^ Christian‐Doppler Laboratory for Portal Hypertension and Liver Fibrosis Medical University of Vienna Vienna Austria; ^4^ Clinical Division of Social Psychiatry, Department of Psychiatry and Psychotherapy Medical University of Vienna Vienna Austria; ^5^ Comprehensive Center for Clinical Neurosciences and Mental Health Medical University of Vienna Vienna Austria; ^6^ Psychosocial Health Center ESRA Vienna Austria; ^7^ Department of Nutritional Sciences, Molecular Nutritional Science University of Vienna Vienna Austria

**Keywords:** alcohol‐associated liver disease, complement, IgM, malondialdehyde

## Abstract

**Background and Aims:**

Alcohol‐related liver disease (ALD) is associated with elevated blood immunoglobulin‐type M (IgM) levels and hepatic lipid peroxidation. Nevertheless, the functional relevance of systemic IgM targeting lipid peroxidation products during ALD is incompletely understood.

**Methods:**

Levels of IgM and IgG recognising malondialdehyde–acetaldehyde (MAA), as a hallmark epitope of lipid peroxidation, as well as complement factors were assessed in the serum of patients with ALD. The influence of alcohol abstinence on anti‐MAA IgM and IgG levels was determined in AUD patients. A chronic‐binge ethanol diet was given to mice deficient in sialic acid‐binding immunoglobulin‐like lectin G (*Siglec‐G* ^*−/−*^), with specifically increased systemic IgM, and mice lacking soluble IgM (*sIgM* ^*−/−*^), and their corresponding littermates. Furthermore, wildtype mice were injected with MAA‐binding IgM antibodies (LR04) or isotype control IgM during chronic‐binge ethanol feeding. *Siglec‐G* ^*−/−*^ bone marrow transplantation into wildtype or complement C3 deficient mice (*C3* ^*−/−*^) was performed to investigate the involvement of complement activation by elevated IgM in ALD.

**Results:**

Serum levels of anti‐MAA IgM positively correlated with ALD severity in humans. While mice lacking soluble IgM had less pronounced liver injury, increased circulating anti‐MAA IgM titers in *Siglec‐G* ^*−/−*^ mice associated with more hepatocellular damage after ethanol feeding than wildtypes. Similarly, mice receiving LR04 displayed elevated liver injury compared to control‐injected mice. Moreover, besides less hepatic neutrophil and macrophage content, and stellate cell activation than wildtypes, ethanol‐fed *Siglec‐G* ^*−/−*^ and LR04‐treated mice had increased hepatic C3b deposition. Mice deficient in C3 displayed ameliorated ethanol‐induced liver injury compared with controls, despite similarly high anti‐MAA IgM levels after *Siglec‐G* ^*−/−*^ bone marrow transplantation, suggesting complement‐dependent liver injury upon high anti‐MAA IgM. In line, levels of MAA‐binding IgM inversely correlated with C3c and C4 in serum of patients with ALD.

**Conclusion:**

Elevated systemic IgM titres that recognise MDA facilitate complement recruitment, which enhances hepatocyte injury, thereby promoting alcohol‐associated liver disease.

**Summary**

- Alcohol‐related liver disease is a major global health problem with limited treatment options.
- Using various mouse models and patient‐derived samples, we found that IgM antibodies can promote liver injury by activating the complement system, a part of the body's immune defence, thereby identifying novel targets to improve therapy.

**Abbreviations**
αSMA: alpha‐smooth muscle actin
ADH1: alcohol dehydrogenase 1
ALD: alcohol‐associated liver disease
ALT: alanine aminotransferase
AST: aspartate aminotransferase
AUD: alcohol use disorder
CCR2: C‐C motif chemokine receptor 2CD11B: Integrin alpha M
CHILD: Child–Pugh score
COL1A1: collagen, Type I, alpha 1
COL3A1: collagen, Type III, alpha 1
CRP: C‐reactive protein
CXCL1: C‐X‐C motif chemokine ligand 1
CYP2E1: cytochrome P450 family 2 subfamily E member 1
EtOH: ethanol
H&E: haematoxylin and eosin
IgA: immunoglobulin Type A
IgM: immunoglobulin Type M
IgG: immunoglobulin Type G
IL‐1β: interleukin 1 beta
IL‐6: interleukin 6
IL‐10: interleukin 10
MAA: malondialdehyde–acetaldehyde
MDA: malondialdehyde
MELD: model for end‐stage liver disease
PC: phosphocholine
TGFβ: transforming growth factor beta

## 1 Introduction

Alcohol‐associated liver disease (ALD) is increasingly being recognised as a significant global health issue and socioeconomic burden, contributing substantially to liver‐related morbidity and mortality [1, 2]. ALD encompasses a wide range of hepatic conditions, from steatosis to fibrosis and end‐stage cirrhosis, which can be divided into compensated and decompensated stages with different clinical features and prognoses [3, 4]. An acute inflammatory condition within the spectrum of ALD is alcoholic hepatitis, which has a mortality rate of up to 30% within 90 days [1]. The current treatment options are suboptimal, requiring liver transplantation for end‐stage liver disease patients [5]. Despite considerable advances in defining novel therapy targets [6], the underlying mechanisms by which alcohol intake contributes to hepatic injury are not fully understood, which is necessary to improve patient outcomes.

The liver is one of the primary organs affected by excessive alcohol intake due to its major role in alcohol (ethanol) metabolism [7, 8, 9]. Under homeostatic conditions, hepatocytes metabolise ethanol to acetaldehyde, subsequently to acetate and then to carbon dioxide and water for elimination. Yet, upon chronic alcohol consumption, this process is disturbed, resulting in the increased accumulation of reactive aldehydes, such as malondialdehyde (MDA), a byproduct of lipid peroxidation during oxidative stress. MDA and acetaldehyde together acetaldehyde react with proteins to form MDA‐ and MAA‐protein adducts [10]. Hence, hepatic MDA and MAA deposition have been shown to be present during human ALD, as well as in rodent models of ALD [11, 12, 13, 14, 15]. Importantly, these protein adducts can provoke immune responses and stimulate cell death, thereby influencing disease progression.

In addition to hepatic MDA accumulation, it has been shown that patients with ALD have increased immunoglobulin‐type G (IgG) titres in circulation that bind MDA and MAA epitopes [16]. Besides IgG, MDA epitopes are recognised by IgM antibodies, which are essential for activating the complement system, neutralising pathogens and clearing apoptotic cells [17, 18, 19]. Earlier findings from others and us indicated that IgM antibodies recognising MDA epitopes reduce hepatic inflammation during metabolic dysfunction‐associated steatotic liver disease (MASLD) [20, 21, 22]. Furthermore, we found that levels of IgM targeting oxidation‐specific epitopes including MDA are reduced during MASLD compared with healthy individuals [23]. Yet, functional studies describing the role of MDA‐specific IgM antibodies in the context of ALD are currently lacking. Here, using patient‐derived samples and murine models, we describe the functional properties of elevated MDA‐recognising IgM antibodies during ALD. These insights are essential for unravelling the complex immunological landscape of ALD and for developing targeted therapeutic strategies to mitigate liver damage and promote tissue repair.

## 2 Results

### 2.1 Systemic IgM Antibody Titres Targeting MDA and MAA Correlate With Liver Disease Severity in Cirrhotic ALD Patients

**FIGURE 1:**
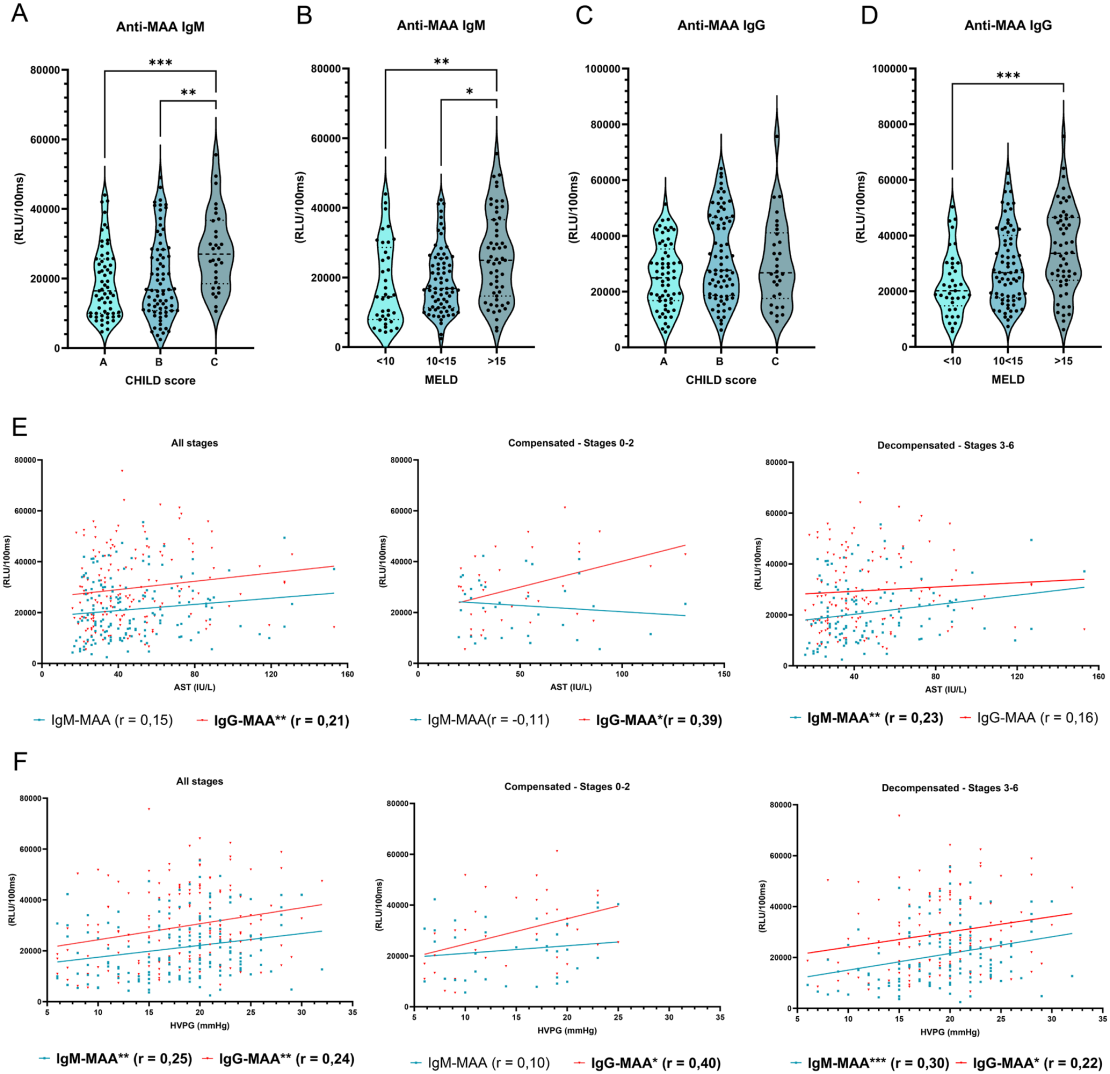
Systemic anti‐MAA IgM and IgG levels during human ALD. (A) Serum anti‐MAA IgM levels in patients with ALD, classified according to the CHILD score. (B) Serum anti‐MAA IgM levels in patients with ALD, classified according to the MELD score. (C) Serum anti‐MAA IgG levels in patients with ALD, classified according to the CHILD score. (D) Serum anti‐MAA IgG levels in patients with ALD, classified according to the MELD score. (E) Correlation analyses between serum AST (IU/L) levels and anti‐MAA IgM and IgG titres in all ALD patients, stratified into compensated or decompensated cirrhosis. (F) Correlation analyses between HVPG (mmHg) measurement and anti‐MAA IgM and IgG titres in all ALD patients, stratified into compensated or decompensated cirrhosis. Data shown as mean ± SEM of *n* = 204 patients. *r* indicates Spearman's correlation coefficient. *p ≤ 0.05, **p ≤ 0.01, ***p ≤ 0.001.

We previously showed that total IgM titres in the blood are increased in patients with advanced chronic liver diseases, including cirrhotic ALD patients [24]. Here, we sought to determine the amount of total IgM and IgG, as well as IgM and IgG titres targeting MAA, which contain MDA epitopes and therefore represent both epitopes, and MDA in the serum of patients diagnosed with ALD, including both decompensated and compensated cirrhotic liver disease stages. In addition to higher total IgM and IgG antibody titres (Figure S1A–D), we found that anti‐MAA IgM antibody levels in the serum of patients with cirrhotic ALD were elevated in individuals with more severe liver disease, as stratification based on the Child–Pugh score (CHILD) and the model for end‐stage liver disease (MELD) score indicated significantly higher anti‐MAA IgM antibody titres between different staging (Figure 1A,B). In contrast, systemic IgG titres to MAA were only significantly higher in patients with more severe liver disease when stratified based on the MELD score (Figure 1C,D). Similar data were obtained for IgM and IgG levels to MDA (Figure S1E–H). Furthermore, correlation analyses revealed that particularly in ALD‐related decompensated cirrhosis, specific IgM titres to MDA and MAA positively correlate with serum AST (IU/L) and hepatic venous pressure gradients (HVPG [mmHg]), a relevant predictor of ALD outcome [25], while a significant correlation was found with IgG levels in the compensated stage (Figure 1E,F and S1I,J; Table S1). No significant correlation was found between MDA‐ and MAA‐binding antibody levels and the inflammatory markers CRP (mg/dL) and IL‐6 (pg/mL) in the serum of ALD patients (Figure S1K–L; Table S1). These data indicate that liver injury and disease severity in human ALD associate with an elevation of MDA‐ and MAA‐binding IgM and IgG antibodies.

**FIGURE 2:**
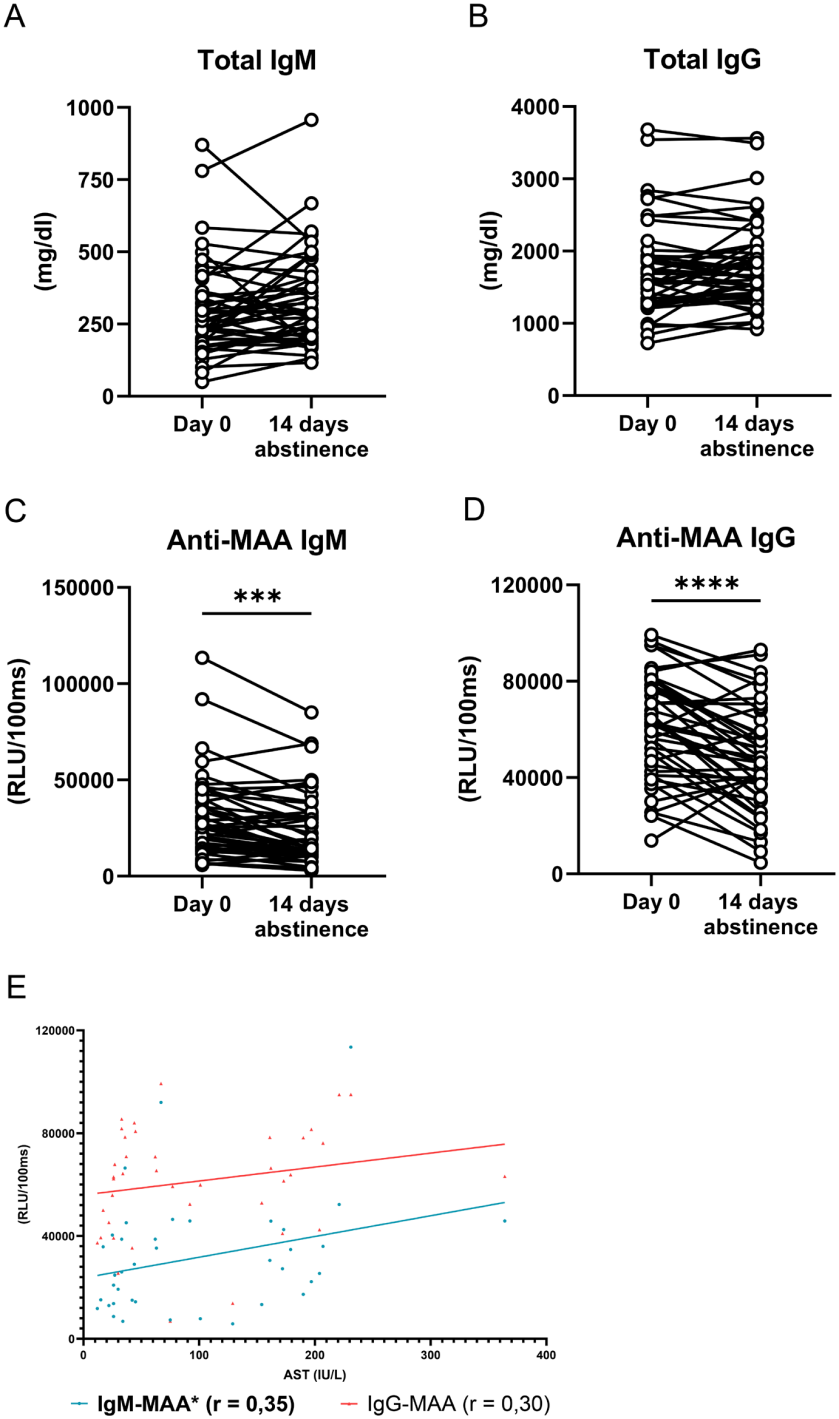
Systemic total and anti‐MAA IgM and IgG levels in AUD patients. (A) Serum IgM levels in patients with AUD at baseline and after 14 days of alcohol abstinence. (B) Serum IgG levels in patients with AUD at baseline and after 14 days of alcohol abstinence. (C) Serum IgM levels in patients with AUD at baseline and after 14 days of alcohol abstinence. (D) Serum IgM levels in patients with AUD at baseline and after 14 days of alcohol abstinence. (E) Correlation analyses between serum AST (IU/L) levels and anti‐MAA IgM and IgG titres in all AUD patients at baseline. Data shown as mean ± SEM of *n* = 50 patients. *r* indicates Spearman's correlation coefficient. *p ≤ 0.05, ***p ≤ 0.001, ****p ≤ 0.0001.

To further evaluate the effect of excessive alcohol consumption on systemic antibody levels towards lipid peroxidation products, we performed a longitudinal analysis of total and MAA‐specific IgM and IgG levels in the serum of patients with alcohol use disorder (AUD) undergoing alcohol withdrawal. We observed a significant decline in anti‐MAA IgM and IgG titers after 14 days of abstinence, despite unchanged total IgM and IgG antibody levels (Figure 2A–D). In line with our findings in ALD patients, correlation analyses revealed that anti‐MAA IgM titres positively correlate with serum AST (IU/L) (Figure 2E; Table S2). This further supports the idea that anti‐MAA antibody elevation is dynamically linked to active alcohol‐induced liver damage.

### 2.2 Mice Deficient in Secreted IgM Develop Less Liver Injury After Chronic–Binge Ethanol Diet

**FIGURE 3:**
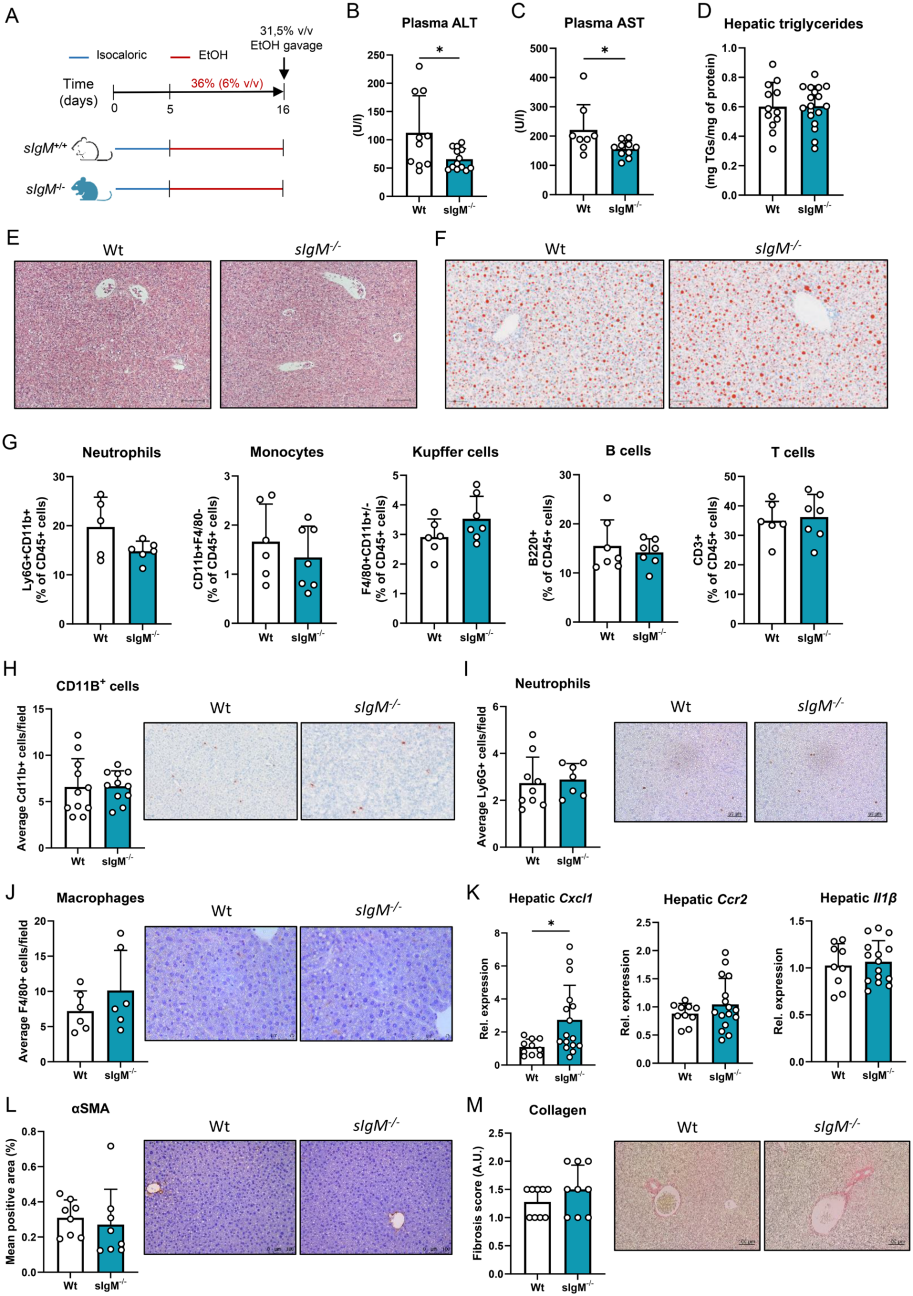
Murine ALD development in *sIgM*‐deficient mice after chronic–binge ethanol feeding. (A) Schematic of the chronic‐binge ethanol feeding study in female sIgM^−/−^ mice (turquoise) and wildtype littermates (white). (B) Plasma ALT levels at the end of the study. (C) Plasma AST levels at the end of the study. (D) Hepatic triglyceride content. (E) Representative images showing H&E staining of liver sections. Scale bar indicates 100 μm. (F) Representative images showing Oil Red O staining of liver sections. Scale bar indicates 100 μm. (G) Flow cytometry analysis of neutrophils (Ly6G^+^), monocytes (CD11B^+^F4/80^−^), Kupffer cells (CD11B^+/−^F4/80^+^), B cells (B220^+^), and T cells (CD3^+^) in the liver. Data are shown relative to the total amount of immune cells present (CD45^+^). (H) Quantification and representative images of immunohistochemical staining for infiltrating macrophages and neutrophils in the liver using Mac‐1. Scale bar indicates 50 μm. (I) Quantification and representative images of immunohistochemical staining for neutrophils in the liver using Ly6G/Ly6C. Scale bar indicates 50 μm. (J) Quantification and representative images of immunohistochemical staining for macrophages in the liver using F4/80. Scale bar indicates 50 μm. (K) mRNA levels of indicated genes (Cxcl1, Ccr2, Il1β) in livers of ethanol‐fed mice, assessed by qPCR. Data are shown relative to wildtype mice and normalised to 18S. (L) Quantification and representative images of immunohistochemical staining for hepatic stellate cell activation using αSMA. Scale bar indicates 100 μm. (M) Quantification and representative images of immunohistochemical staining for hepatic fibrosis using Sirius red. Scale bar indicates 100 μm. Data shown as mean ± SEM of *n* = 10–17/group (B–D, K) or *n* = 5–10/group (G–J, L, M). *p ≤ 0.05.

At first, to assess the contribution of IgM antibodies to ALD, the chronic‐binge ethanol feeding model, 10 days Lieber‐DeCarli diet (6% EtOH v/v) followed by a single binge of ethanol (31.5% v/v), was applied to *sIgM* ^*−/−*^ mice and their wildtype (Wt) littermates (Figure 3A). Consistent with previously described results concluding that *sIgM*‐deficiency protects from early ethanol‐induced liver damage after short‐term ethanol diet [26], we found that chronic‐binge ethanol diet resulted in lower plasma ALT and AST in *sIgM* ^*−/−*^ mice compared with their controls (Figure 3B,C). Furthermore, we did not observe any alterations in general liver morphology, liver‐to‐body weight ratio and hepatic triglyceride content (Figures 3D–F and S3A). Hepatic profiling of major immune cell populations with flow cytometry and immunohistochemistry did not reveal differences in the amount of neutrophils, monocytes, macrophages, B cells, T cells, CD4 and CD8 T cells, natural killer cells and dendritic cells between *sIgM* ^*−/−*^ mice and Wt controls after ethanol diet (Figures 3G–J and S2B–E). In line, gene expression analyses indicated similar mRNA levels of *Ccr2*, *Il1β* and *Il10*, despite a small increase in *Cxcl1* (Figures 3K and S2F). Moreover, we did not find altered αSMA levels or collagen deposition in the livers, suggesting unaffected fibrosis development in *sIgM* ^*−/−*^ mice after chronic‐binge ethanol diet, confirmed by similar gene expression levels of *Col1a1*, *Col3a1* and slightly increased *Tgfβ* (Figures 3L,M and S2G–I). Plasma ethanol levels and hepatic expression of *Cyp2e1* and *Adh1* mRNAs, which regulate the hepatic metabolism of ethanol, were unchanged between Wt and *sIgM* ^*−/−*^ mice (Figure S2J–L), indicating that the lack of IgM antibodies does not affect ethanol absorption and metabolism. Notably, mice deficient in sIgM displayed elevated levels of IgA in circulation, while plasma IgG1 and anti‐MDA IgG1 titres were reduced after ethanol diet (Figure S2M–O), thereby potentially conferring effects on ethanol‐related liver injury, independent of IgM‐related mechanisms.

### 2.3 Elevated Systemic IgM Titres in Mice Lacking Siglec‐G Associates With More Ethanol‐Induced Hepatocyte Injury

In line with our observations in ALD and AUD patients, we found that murine ALD in mice on the chronic‐binge ethanol feeding model associates with elevated IgM titres to MDA and MAA compared to isocaloric control‐fed mice when normalised to total plasma IgM levels (Figure S3A,B), suggesting a unique expansion and prominent role of IgM towards these epitopes upon alcohol consumption in mice.

**FIGURE 4:**
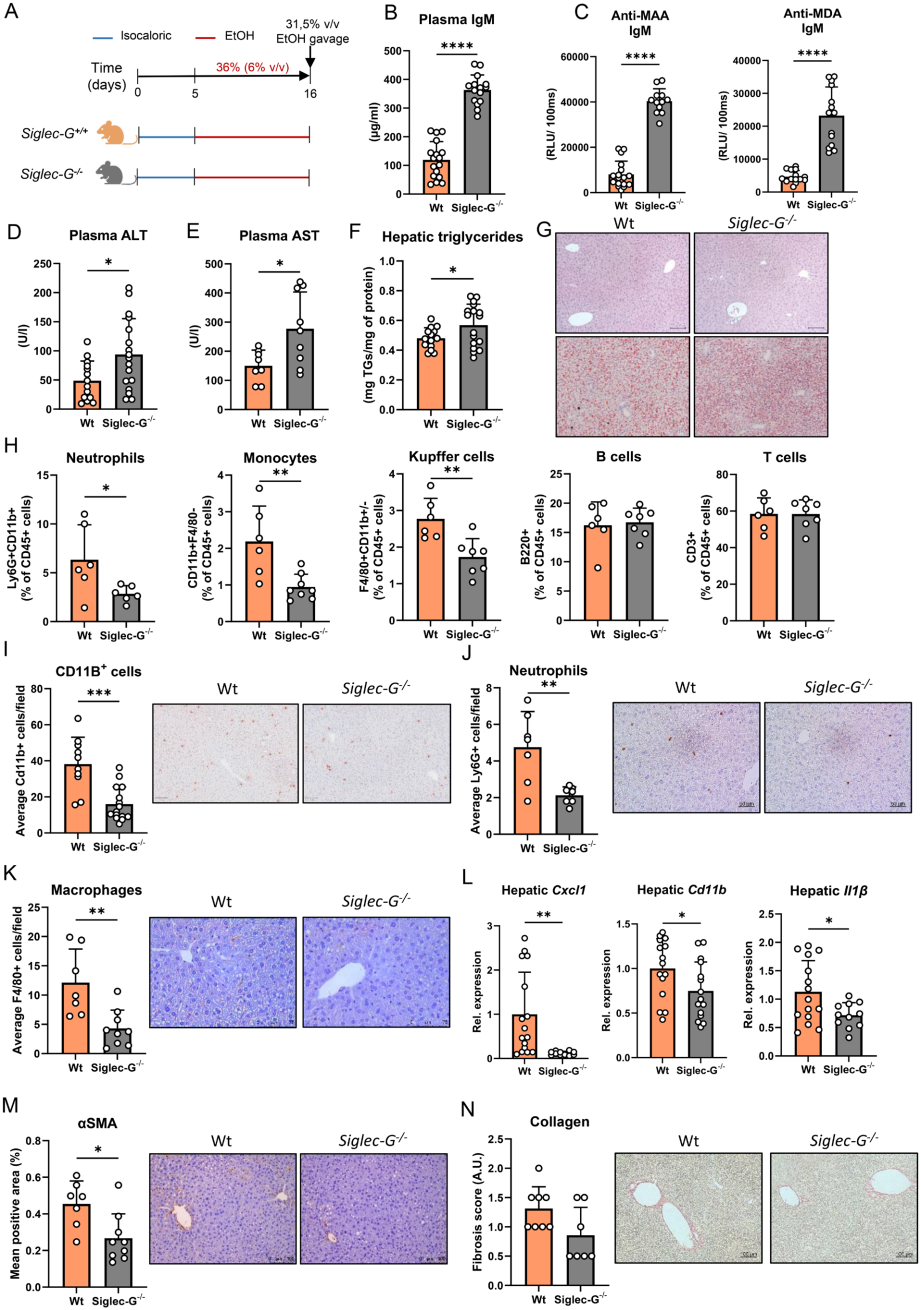
Increased systemic anti‐MAA IgM levels during Siglec‐G deficiency mediates ethanol‐induced liver injury. (A) Schematic of chronic‐binge ethanol feeding study in female Siglec‐G^−/−^ mice (grey) and wildtype littermates (orange). (B) Plasma IgM levels at the end of the study. (C) Plasma anti‐MAA and anti‐MDA IgM levels at the end of the study. (D) Plasma ALT levels. (E) Plasma AST levels. (F) Hepatic triglyceride content. (G) Representative images showing H&E and Oil Red O staining of liver sections. Scale bars indicate 100 μm. (H) Flow cytometry analysis of neutrophils (Ly6G^+^), monocytes (CD11B^+^F4/80^−^), Kupffer cells (CD11B^+/−^F4/80^+^), B cells (B220^+^) and T cells (CD3^+^) in the liver. Data are shown relative to the total amount of immune cells present (CD45^+^). (I) Quantification and representative images of immunohistochemical staining for infiltrating macrophages and neutrophils in the liver using Mac‐1. Scale bar indicates 100 μm. (J) Quantification and representative images of immunohistochemical staining for neutrophils in the liver using Ly6G/Ly6C. Scale bar indicates 50 μm. (K) Quantification and representative images of immunohistochemical staining for macrophages in the liver using F4/80. Scale bar indicates 50 μm. (L) mRNA levels of indicated genes (Cxcl1, Cd11b, Il1β) in livers of ethanol‐fed mice, assessed by qPCR. Data are shown relative to wildtype mice and normalised to 18S. (M) Quantification and representative images of immunohistochemical staining for hepatic stellate cell activation using αSMA. Scale bar indicates 100 μm. (N) Quantification and representative images of immunohistochemical staining for hepatic fibrosis using Sirius red. Scale bar indicates 100 μm. Data shown as mean ± SEM of *n* = 17–16/group (B–F, L) or *n* = 6–10/group (H–K, M, N). *p ≤ 0.05, **p ≤ 0.01, ***p ≤ 0.001, ****p ≤ 0.0001.

Hence, to study the direct functional consequences of high systemic anti‐MAA IgM levels in ALD, in complement to using *sIgM* ^*−/−*^ mice, mice lacking the sialic acid‐binding immunoglobulin‐type lectin‐G (*Siglec‐G* ^*−/−*^) were used. Owing to the lack of the inhibitory coreceptor, especially on B1 cells, *Siglec‐G* ^*−/−*^ mice are characterised by a robust increase in total circulatory IgM [27]. Moreover, others and we have previously shown that *Siglec‐G* deficiency in mice results in an expansion of IgM antibodies specifically targeting oxidation‐specific epitopes, including MDA and MAA [22], thereby providing an optimal model to investigate the direct influence of high MAA‐IgM on ethanol‐related liver disease development. As expected, isocaloric diet‐fed *Siglec‐G* ^*−/−*^ mice had significantly increased total as well as specific anti‐MAA and anti‐MDA IgM titres in circulation compared with Wt littermates, while total IgG1, anti‐MAA IgG1, anti‐MDA IgG1 and total systemic IgA levels were not different (Figure S4A–G). Importantly, even in the presence of ethanol intake after the chronic‐binge feeding model, *Siglec‐G* deficiency in mice only resulted in an increase in total, as well as MAA‐ and MDA‐specific IgM titres, which associated with more hepatic IgM deposition (Figures 4A–C and S5A), while plasma IgA, IgG1 and anti‐MDA IgG1 titers remained similar (Figure S5B–D).

Elevated anti‐MAA IgM titres in *Siglec‐G* ^*−/−*^ mice associated with increased levels of plasma ALT and AST, indicating more ALD‐associated liver injury (Figure 4D,E). Furthermore, despite comparable liver weights (Figure S5E), *Siglec‐G* ^*−/−*^ mice developed slightly more hepatic steatosis compared to Wt littermates after chronic‐binge ethanol feeding, potentially due to altered lipid handling as a result of hepatocyte injury (Figure 4F,G). Interestingly, *Siglec‐G* ^*−/−*^ mice displayed reduced recruitment of neutrophils and monocytes, and fewer macrophages in the liver (Figures 4H–K and S5F–I), which is associated with less hepatic *Cxcl1*, *Cd11b* and *Il1β* gene expression, while *Tgfβ* and *Il10* remained unchanged (Figures 4L and S5J,K). Moreover, *Siglec‐G* ^*−/−*^ mice had less hepatic stellate cell activation, assessed by αSMA and, slightly less collagen deposition, assessed by Sirius red staining, which might be related to less profibrotic signalling from macrophages (Figure 4M,N). No differences were observed in plasma ethanol concentrations and hepatic mRNA levels of *Cyp2e1* and *Adh1* between ethanol‐fed *Siglec‐G* ^*−/−*^ and Wt mice (Figure S5L–N), suggesting that high IgM antibody levels during *Siglec‐G* deficiency did not affect ethanol absorption and metabolism. Taken together, these data indicate that elevated IgM recognising MDA and MAA during *Siglec‐G* deficiency enhances ethanol‐induced hepatocyte injury that is associated with impaired macrophage function and less inflammatory cell recruitment.

### 2.4 Treatment With MAA‐Binding IgM Antibodies Recapitulates Findings in Mice With Elevated IgM During Siglec‐G‐Deficiency

**FIGURE 5:**
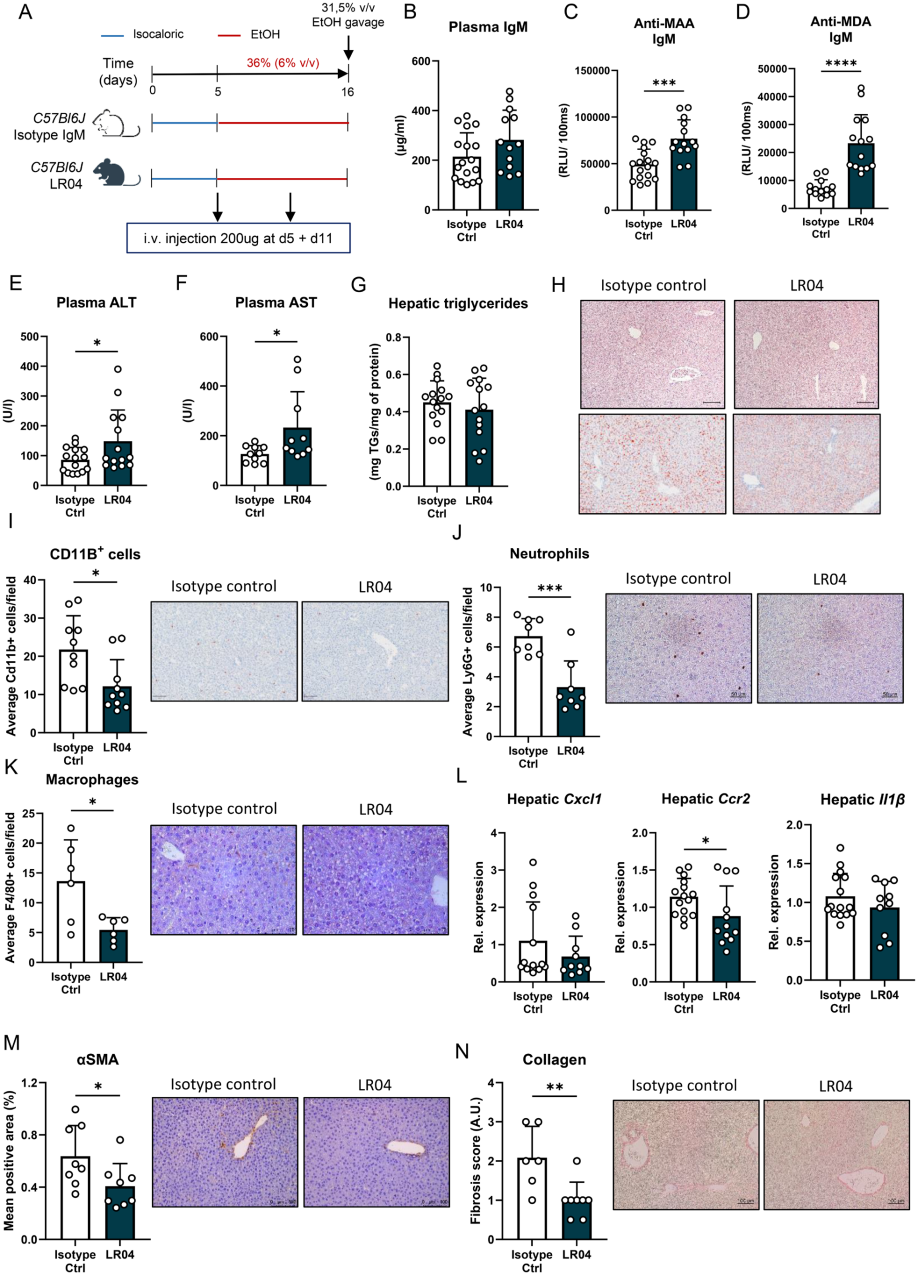
Exogenous MAA‐IgM alters ethanol‐induced liver injury in mice. (A) Schematic of intervention with LR04 antibody (dark green) or isotype control IgM (white) during chronic‐binge ethanol feeding in wildtype female mice. (B) Plasma IgM levels at the end of the study. (C) Plasma anti‐MAA IgM levels at the end of the study. (D) Plasma anti‐MDA IgM levels at the end of the study. (E) Plasma ALT levels. (F) Plasma AST levels. (G) Hepatic triglyceride content. (H) Representative images showing H&E and Oil Red O staining of liver sections. Scale bar indicates 100 μm. (I) Quantification and representative images of immunohistochemical staining for infiltrating macrophages and neutrophils in the liver using Mac‐1. Scale bar indicates 100 μm. (J) Quantification and representative images of immunohistochemical staining for neutrophils in the liver using Ly6G/Ly6C. Scale bar indicates 50 μm. (K) Quantification and representative images of immunohistochemical staining for macrophages in the liver using F4/80. Scale bar indicates 50 μm. (L) mRNA levels of indicated genes (Cxcl1, Ccr2, Il1β) in livers of ethanol‐fed mice, assessed by qPCR. Data are shown relative to wildtype mice and normalised to 18S. (M) Quantification and representative images of immunohistochemical staining for hepatic stellate cell activation using αSMA. Scale bar indicates 100 μm. (N) Quantification and representative images of immunohistochemical staining for hepatic fibrosis using Sirius red. Scale bar indicates 100 μm. Data shown as mean ± SEM of *n* = 17–13 mice/group (B–H, L) or *n* = 6–10 mice/group (I–K, M, N). *p ≤ 0.05, **p ≤ 0.01, ***p ≤ 0.001, ****p ≤ 0.0001.

To further evaluate the direct contribution of MAA‐targeting IgM antibodies to ethanol‐induced liver disease in mice, MDA‐ and MAA‐specific IgM (LR04; 200 μg) or isotype control IgM was administered twice to female wildtype mice via tail vein injection during the chronic‐binge ethanol feeding model; at the start of ethanol feeding and 5 days later (Figure 5A). Similar to our previous study [20], this approach was sufficient to specifically elevate systemic anti‐MAA and anti‐MDA IgM antibody titres in LR04‐treated mice, while the total amount of IgM was similar (Figure 5B–D). Systemic IgA as well as the amount of IgM binding to phosphorylcholine (PC), levels of total IgG1 and IgG1 binding to MAA and MDA were not different from those in mice injected with isotype control IgM, indicating the specificity of LR04 (Figure S6A–E).

While LR04 treatment during ethanol feeding did not alter the liver‐to‐body weight ratio or hepatic triglyceride levels, plasma levels of ALT and AST were significantly higher than in controls (Figures 5E–H and S6F). In line with our observations in *Siglec‐G* ^*−/−*^ mice, histological assessment indicated that elevated anti‐MAA IgM levels in LR04‐treated mice resulted in fewer macrophages and neutrophils in the liver compared with isotype control‐treated mice, confirmed by reduced mRNA levels of *Ccr2* and *Ly6g*, while *Cxcl1* showed a reducing trend, *Il1β* was unchanged, and *Il10* slightly increased (Figures 5L and S6G,H). Moreover, administration of LR04 resulted in less hepatic stellate cell activation (αSMA), collagen deposition, and reduced *Col3a1* expression (Figures 5M,N and S6I–J), suggesting less fibrosis development, as observed in *Siglec‐G* ^*−/−*^ mice. Plasma ethanol concentrations and hepatic mRNA levels of *Adh1* and *Cyp2e1* were comparable to control‐treated mice (Figure S6K–M). These data further support that IgM antibodies that recognise MDA and MAA epitopes are functionally contributing to hepatocellular damage during ALD, complementing our findings in mice that have high anti‐MAA IgM due to the lack of Siglec‐G.

### 2.5 High Anti‐MAA IgM Titers Promote Ethanol‐Related Liver Injury in a Complement‐Dependent Fashion

**FIGURE 6:**
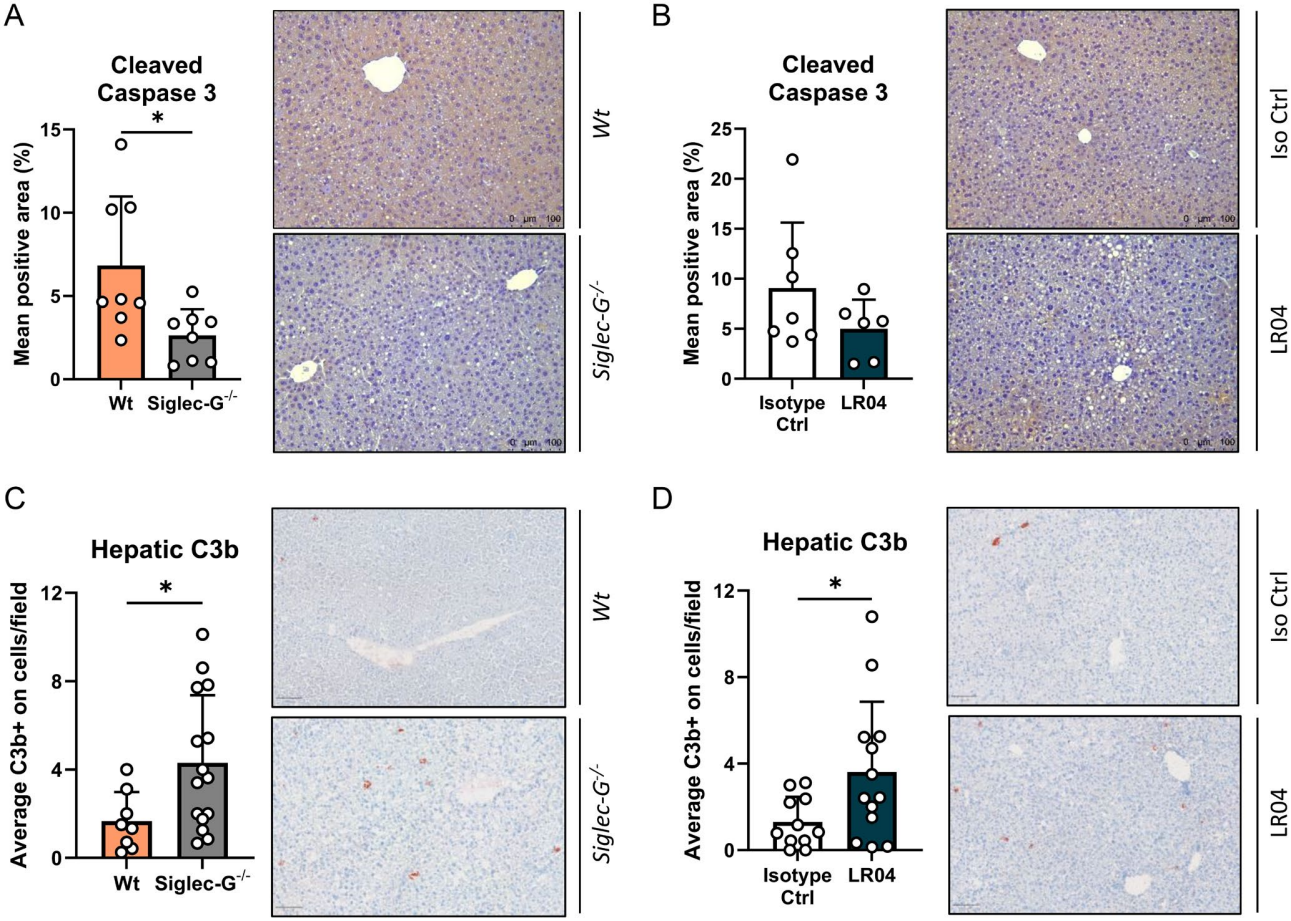
Increased hepatic C3b deposition in mice with high systemic anti‐MAA IgM. (A) Quantification and representative images of immunohistochemical staining for cleaved caspase 3 in the livers of ethanol‐fed Wt and Siglec‐G^−/−^ mice, as shown in Figure 4A. Scale bar indicates 100 μm. (B) Quantification and representative images of immunohistochemical staining for cleaved caspase 3 in the liver of ethanol‐fed mice treated with isotype control IgM or LR04, as shown in Figure 5A. Scale bar indicates 100 μm. (C) Quantification and representative images of immunohistochemical staining for complement C3b in the livers of ethanol‐fed Wt and Siglec‐G^−/−^ mice, as shown in Figure 4A. Scale bar indicates 100 μm. (D) Quantification and representative images of immunohistochemical staining for complement C3b in the liver of ethanol‐fed mice treated with isotype control IgM or LR04, as shown in Figure 5A. Scale bar indicates 100 μm. Data shown as mean ± SEM of *n* = 8 mice/group (A, B), *n* = 8–15 mice/group (C), or *n* = 11–13 mice/group (D). *p ≤ 0.05.

Next, we determined whether liver injury during high anti‐MAA IgM titres relates to altered hepatocyte apoptosis. *Siglec‐G* ^*−/−*^ mice had significantly lower levels of cleaved caspase 3 compared to wildtypes after ethanol diet, while a similar trend was observed in LR04‐treated mice compared with their controls (Figure 6A,B), suggesting that apoptotic cell death is not the primary cause of elevated liver damage. Yet, given that IgM antibodies can recruit and activate the complement system, which would primarily result in necrosis and complement‐mediated lytic cell death, we determined hepatic C3b accumulation. Both *Siglec‐G* ^*−/−*^ and LR04‐treated mice showed increased deposition of C3b in the liver compared to their respective controls after a chronic‐binge ethanol diet (Figure 6C,D), suggesting that elevated levels of MAA‐binding IgM in murine alcohol‐associated liver injury are associated with complement recruitment and activation in the liver.

**FIGURE 7:**
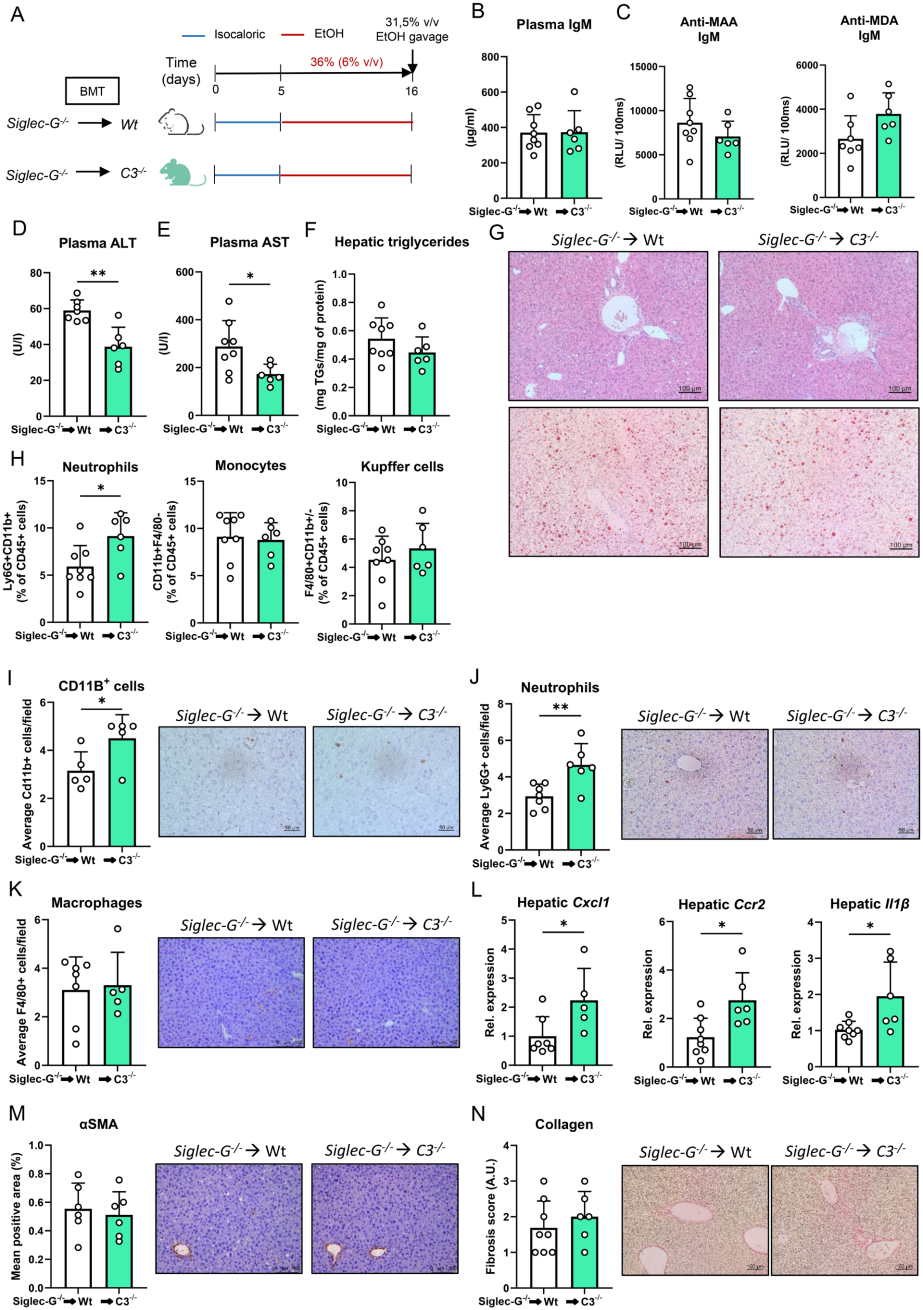
Lack of C3 reverses effects on ALD mediated by increased systemic anti‐MAA IgM levels during Siglec‐G deficiency. (A) Schematic of chronic‐binge ethanol feeding study in female Wt (white) and C3^−/−^ (light green) mice that were transplanted with bone marrow isolated from Siglec‐G^−/−^ mice. (B) Plasma IgM levels at the end of the study. (C) Plasma anti‐MAA and anti‐MDA IgM levels at the end of the study. (D) Plasma ALT levels. (E) Plasma AST levels. (F) Hepatic triglyceride content. (G) Representative images showing H&E and Oil Red O staining of liver sections. Scale bars indicate 100 μm. (H) Flow cytometry analysis of neutrophils (Ly6G^+^), monocytes (CD11B^+^F4/80^−^) and Kupffer cells (CD11B^+/−^F4/80^+^) in the liver. Data are shown relative to the total amount of immune cells present (CD45^+^). (I) Quantification and representative images of immunohistochemical staining for infiltrating macrophages and neutrophils in the liver using Mac‐1. Scale bar indicates 100 μm. (J) Quantification and representative images of immunohistochemical staining for neutrophils in the liver using Ly6G/Ly6C. Scale bar indicates 50 μm. (K) Quantification and representative images of immunohistochemical staining for macrophages in the liver using F4/80. Scale bar indicates 50 μm. (L) mRNA levels of indicated genes (Cxcl1, Ccr2, Il1β) in livers of ethanol‐fed mice, assessed by qPCR. Data are shown relative to wildtype mice and normalised to Cyclophilin B. (M) Quantification and representative images of immunohistochemical staining for hepatic stellate cell activation using αSMA. Scale bar indicates 100 μm. (N) Quantification and representative images of immunohistochemical staining for hepatic fibrosis using Sirius red. Scale bar indicates 100 μm. Data shown as mean ± SEM of *n* = 8–6/group. *p ≤ 0.05, **p ≤ 0.01.

While *C3* ^*−/−*^ mice lack the central component of complement activation, these mice do not have altered systemic amounts of IgM and anti‐MDA and ‐MAA IgM after chronic‐binge ethanol diet (Figure S7A–C). Moreover, we did not observe any differences in the development of ethanol‐induced steatohepatitis and liver injury, nor in hepatic ethanol metabolism in *C3* ^*−/−*^ mice compared to Wt mice (Figure S7D–P). To identify whether the observed effects of anti‐MAA IgM during *Siglec‐G* deficiency depend on complement‐mediated mechanisms, bone marrow from *Siglec‐G* ^*−/−*^ mice was transplanted into female Wt or *C3* ^*−/−*^ recipient mice. After a 6 week recovery period, the recipient mice were fed an ethanol diet according to the chronic‐binge feeding model (Figure 7A). Importantly, despite the fact that *Siglec‐G* deficiency resulted in similarly high levels of total IgM in circulation, as well as specific IgM recognising MDA and MAA (Figure 7B,C), *C3* ^*−/−*^ recipient mice developed less ethanol‐induced hepatic injury compared with Wt mice, as shown by reduced plasma ALT and AST levels (Figure 7D,E). Hepatic steatosis and triglyceride content, as well as plasma IgG1 and anti‐MDA IgG1 titres did not differ between the groups (Figures 7F,G and S8A,B). Opposing liver injury markers, recipient mice lacking *C3* had more neutrophils in the liver and elevated expression of *Cxcl1*, *Ccr2* and *Il1β*, in addition to the complete absence of *C3* (Figures 7H–L and S8C). Levels of αSMA and collagen deposition in the liver, as well as hepatic mRNA levels of *Il10*, *Tgfβ*, *Col3a1*, *Cyp2e1* and *Adh1* were unchanged between Wt and *C3* ^*−/−*^ recipient mice, indicating no difference in fibrosis and ethanol metabolism (Figure S8D–H). Taken together, these data suggest that elevated levels of IgM antibodies that specifically recognise MDA and MAA epitopes can promote liver injury during ALD via enhanced complement activation.

### 2.6 Anti‐MAA IgM Titres Inversely Correlate With Serum Complement Factors in Human ALD

**FIGURE 8:**
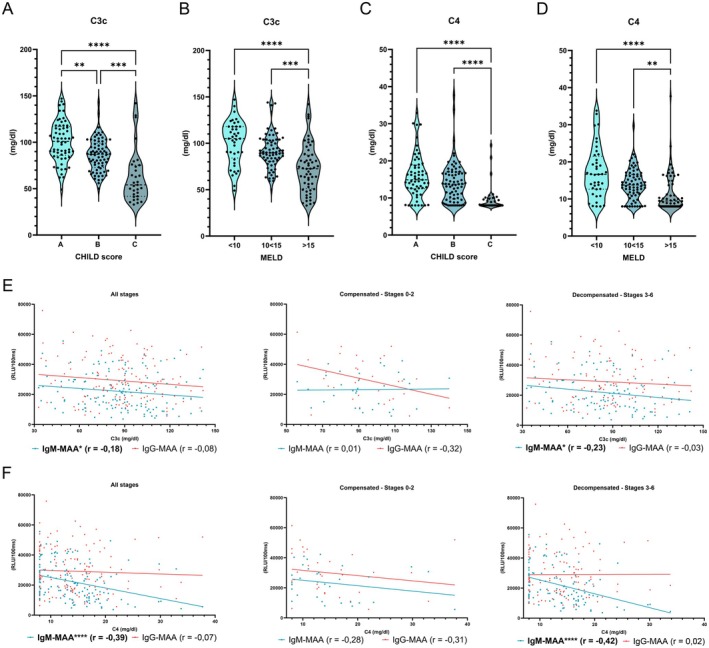
Systemic anti‐MAA IgM correlates with altered complement factors in ALD patients. (A) Serum C3c levels in patients with ALD, classified according to the CHILD score. (B) Serum C3c levels in patients with ALD, classified according to the MELD score. (C) Serum C4 levels in patients with ALD, classified according to the CHILD score. (D) Serum C4 levels in patients with ALD, classified according to the MELD score. (E) Correlation analyses between serum C3c (mg/dL) and anti‐MAA IgM and IgG titres in ALD patients, stratified into compensated or decompensated cirrhosis. (F) Correlation analyses between serum C4 (mg/dL) and anti‐MAA IgM and IgG titres in ALD patients, stratified into compensated or decompensated cirrhosis. Data shown as mean ± SEM of *n* = 204 patients. *r* indicates Spearman's correlation coefficient. **p ≤ 0.01, ***p ≤ 0.001, ****p ≤ 0.0001.

Given our findings in our murine models, we assessed the serum complement factors C3c (mg/dL) and C4 (mg/dL) in our human ALD cohort, which we previously found to be inversely correlating with disease severity and systemic inflammation in advanced chronic liver disease patients [24]. Similarly, we observed that serum C3c and C4 levels are lower in ALD patients with a higher CHILD and MELD score (Figure 8A–D). Especially in decompensated cirrhosis, we found that anti‐MAA IgM, and to a lesser extent also anti‐MDA IgM levels, negatively correlated with complement factors C3c and C4, while no correlation was observed with anti‐MAA IgG titres (Figures 8E,F and S9A,B; Table S1). Taken together, these data further support that elevated anti‐MAA IgM in circulation results in increased complement consumption by the liver, thereby potentially contributing to hepatic injury and accelerated hepatocellular cell death via MAC formation. As such, systemic IgM levels, particularly those binding to MAA, and complement factors are complementarily regulated in human ALD and modulate underlying disease processes and progression.

## 3 Discussion

The current increasing rates of excessive alcohol consumption have generated a continuous rise in the prevalence of alcohol‐associated liver disease, making it one of the most common causes of chronic liver disease globally. ALD is the primary cause of liver‐related mortality worldwide, in which severe alcoholic hepatitis has a particularly high short‐term mortality rate, with 28‐day mortality rates ranging from 20% to 50%, depending on the severity and presence of complications such as renal failure [4]. However, the factors and mechanisms driving the pathogenesis and progression of alcohol‐associated steatohepatitis remain poorly understood [28]. Consequently, suitable preventive and therapeutic strategies, other than abstinence, are currently unavailable, making alcohol‐associated liver cirrhosis the leading indication for liver transplantation [29, 30, 31]. Even with transplantation, the 5‐year survival rate can be lower than that of non‐ALD‐related cirrhosis owing to the risk of alcohol relapse and other complications. Patients with ALD exhibit distinct immunological characteristics due to chronic exposure to alcohol and its metabolites, which impact the immune system and contribute to liver damage [32]. Here, we investigated the contribution of elevated IgM in circulation, particularly IgM antibodies recognising MAA epitopes, to ALD using various murine models and human samples. We found that anti‐MAA IgM antibodies are more prominent in the serum of patients with more severe ALD, correlate with altered complement factors, and modulate liver injury during murine ALD via complement activation.

The role of IgM antibodies in chronic liver disease appears to be multifaceted, involving clearance of oxidised lipids and cellular debris, modulation of inflammatory responses, and activation of the complement system. In ALD, IgM or components of the IgM response (such as complement C1q) have been suggested to exacerbate liver injury and inflammation [33]. Nevertheless, to date, the role of IgM antibodies in ALD has only been studied in mice lacking soluble IgM [26], which display compensatory IgG and IgA levels that might contribute to pathogen neutralisation and immune complex clearance [34]. Because of its importance in regulating mucosal protection against invading pathogens during ethanol‐induced gut leakiness [35], enhanced IgA‐mediated immunity in the intestines may contribute to altered ALD development in *sIgM* ^*−/−*^ mice. In this regard, *Siglec‐G* ^*−/−*^ mice, which lack a negative regulator of the B‐1a cell population, are of particular interest and are favourable for investigating the contribution of elevated IgM to ALD, as they have a robust and specific increase in total plasma IgM due to the expansion of B‐1a cells [27]. Importantly, our findings in *Siglec‐G* ^*−/−*^ mice further support that IgM‐mediated immune mechanisms contribute to ethanol‐associated liver damage. In line, both experimental models and clinical observations from others and our group suggest that IgM may have an important role in MASLD, limiting hepatic inflammation during hyperlipidaemia [20, 21, 22]. In agreement with our findings in MASLD, we observed fewer infiltrating monocytes and neutrophils in ethanol‐fed mice that exhibited increased anti‐MAA IgM titres [17]. Hence, our data suggest that IgM‐mediated ethanol‐related hepatocellular injury, resulting in elevated systemic levels of liver enzymes, occurs independently of the recruitment of infiltrating immune cells, or even more, is a direct consequence of dysfunctional macrophage responses, such as clearance of cellular debris. Further supporting this, we found that a distinct expansion of IgMs with specificity for MAA epitopes, which are known to be present on dying hepatocytes during ALD, is sufficient to increase ethanol‐induced liver injury. Importantly, systemic anti‐MAA IgM titres correlated with disease severity and liver injury in human cirrhotic ALD. As such, excessive accumulation of MDA and MAA on hepatocytes might be a key driver of IgM‐mediated liver injury during ALD, making these epitopes an interesting target with potentially beneficial therapeutic effects. Notably, our data suggest that anti‐MAA IgM is primarily modestly correlated with AST at later disease stages, while IgG titres seem more correlated with AST at earlier stages, suggesting a different immunological dynamic. IgG is a class‐switched, affinity‐matured isotype that reflects a memory‐driven immune response. Therefore, the observed correlation patterns in compensated ALD might be explained by mitochondrial damage and oxidative stress that may not elicit a strong IgM response but could gradually induce IgG class switching. In the decompensated stage, progressive immune dysfunction a higher intensity and frequency of inflammation episodes that may trigger acute‐phase IgM antibodies against oxidation‐specific epitopes (such as MAA) that rise in tandem with AST, while immune dysfunction may impair class switching to IgG antibodies. Alternatively, IgG antibodies might reflect different mechanisms of immune activation or exposure to neoantigens at early stages. We acknowledge that these dynamics warrant further investigation to fully understand the potential different roles of IgM and IgG in ALD pathogenesis.

Our results also indicate that systemic anti‐MAA IgM levels correlate with altered complement factors in circulation and IgM‐mediated liver damage occurs via complement activation. Since the liver is the main source of most soluble complement components, previous studies have focused on unravelling the role of complement in chronic liver disease, including ALD (reviewed in [33]). In the livers of alcohol‐associated hepatitis patients, increased cleavage of C3 into C3b, iC3b and C3c fragments and increased *C1qR*, *C3aR*, *C5aR*, *C5L2* mRNA expression levels were observed [36], indicating alterations in the complement cascade and suggesting its relevance during ALD. Systemically, despite studies reporting no changes compared with control groups [37, 38], the serum concentrations of various complement factors were found to be altered in human ALD. C3 levels were found to be lower, while C1q and C4d/C4 ratios were elevated in human ALD compared to healthy individuals, suggesting an indication of classical pathway activation [39]. These results were confirmed in other studies reporting diminished C4 levels in a group of patients with ALD and severe cirrhotic alcohol‐associated patients [40, 41], supporting our findings that high MAA‐IgM titres negatively correlate with C4 and result in classical complement activation. In addition to these indications in human ALD, studies using animal models have shown that complement activation is involved in ethanol‐induced liver disease progression. Deposition of complement components in the liver after ethanol feeding was reported [42], while mice deficient in certain complement factors developed less ethanol‐induced steatosis and liver damage compared with their wildtype controls [43, 44, 45], making complement an interesting target to improve therapy options. Therefore, complement protein inhibitors were tested in ethanol‐fed mice as a possible treatment for experimental ALD. Administration of C1‐INH (Cinryze) resulted in complement inhibition, which was associated with diminished liver damage and inflammation, supporting its role as a therapeutic intervention [26]. As such, our data suggest that targeting the IgM‐complement system might be an alternative approach to modulate liver injury and hepatocyte function in ALD.

In conclusion, while alterations in serum immunoglobulin levels are characteristic of human patients with chronic liver disease, our novel mechanistic data indicate that increased exposure of MDA on hepatocytes associates with high IgM titres binding these epitopes, which facilitate the activation of the classical complement pathway, thereby promoting liver injury in alcohol‐associated liver disease.

## 4 Methods

### 4.1 Human ALD Cohort

The human cohort included 204 patients with ALD cirrhosis (including MetALD) undergoing minimally invasive HVPG measurement at the Hepatic Hemodynamic Lab at the Vienna General Hospital between February 2019 and September 2021 (Vienna Cirrhosis Study VICIS; NCT03267615) [46]. Patient characteristics are described in the table below. HVPG measurements were conducted in accordance with a standardised protocol, as previously described [47], and the presence of ACLD is defined by the presence of portal hypertension (i.e., an HVPG of ≥ 6 mmHg [46, 48]). Central venous blood samples were collected from the right internal jugular vein for subsequent analyses at the timepoint of HVPG measurement. Besides the exclusion of patients with non‐ALD aetiologies of cirrhosis (viral hepatitis, autoimmune hepatitis, cholestatic liver disease), clinical exclusion criteria were acute decompensation, acute‐on‐chronic liver failure, active infection, a history of transjugular intrahepatic portosystemic shunt (TIPS) insertion or liver transplantation, current or previous PVT and HCC or active nonhepatic malignancies and nonselective beta‐blocker intake at the time of HVPG measurement. All analyses were conducted in accordance with the 1964 Declaration of Helsinki and its later amendments and approved by the local ethics committee of the Medical University of Vienna (2317/2019). All patients signed an informed consent form to participate in the VICIS study.

Blood samples were taken from the catheter introducer sheath at the time point of HVPG measurement, according to standardised and ISO‐certified procedures. Laboratory personnel were blinded to the clinical and haemodynamic data. Quantitative determination of human complement factors C3c (normal range 90–180 mg/dL) and C4 (normal range 10–40 mg/dL), immunoglobulin subtypes IgG (normal range 700–1600 mg/dL), IgM (normal range 40–230 mg/dL) and IgA (normal range 70–400 mg/dL) was performed by nephelometry on a BNII System using N antisera (Siemens Healthcare Diagnostics, Vienna, Austria).

**Table:** 

	Included ALD cohort
	*n* = 204
Age (years)	57.4 (51.6–65.7)
Sex (male)	150 (73.5%)
HVPG (mmHg)	19 (15–22)
Child–Pugh stage	
A	72 (35.5%)
B	93 (45.8%)
C	38 (18.7%)
MELD	13 (10–17)
Albumin (g/L)	35.4 (31.7–38.9)
Platelet count (G/L)	106 (79–144)
AST (U/L)	41 (29–58)
ALT (U/L)	26 (19–35)
GGT (U/L)	107 (57–214)
ALP (U/L)	100 (79–128)
White blood cell count (G/L)	5.11 (3.88–6.43)
CRP (mg/dL)	0.42 (0.20–0.90)
IL‐6 (pg/mL)	12.2 (7.1–24.5)
LBP (μg/mL)	7.10 (5.37–9.37)
IgM (mg/dL)	125.5 (87.625–215)
IgG (mg/dL)	1410 (1127.5–1832.5)
IgA (mg/dL)	481 (325–676.5)

### 4.2 Human AUD Cohort

This study was conducted at the Department for Psychiatry and Psychotherapy of the Medical University of Vienna (Austria) between November 2019 and April 2021. Patients admitted for in‐patient alcohol withdrawal treatment were screened consecutively for inclusion and exclusion criteria upon consent to participate. To be eligible for enrolment, patients had to meet diagnostic criteria of alcohol dependence according to ICD‐10 (code F10.2), be between 18 and 65 years of age, and have adequate language skills. Exclusion criteria comprised the presence of a condition accompanied by impaired cognitive functioning, such as any type of dementia, other neurodegenerative diseases or specific psychiatric conditions, such as severe depression with or without psychotic symptoms, schizophrenia spectrum and other psychotic disorders (ICD‐10 F2.X). Additionally, patients with ongoing 5‐fluorouracil treatment, a medical history of bariatric surgery or previously diagnosed eating disorders (i.e., anorexia nervosa or bulimia nervosa) were excluded from the study. Furthermore, patients who had received any form of thiamine substitution (TS) within the last 4 weeks were also excluded from this study. Following the standard of care, patients were required to remain abstinent throughout the whole in‐patient stay, and withdrawal symptoms were treated with oxazepam in a stepwise reduction. Abstinence was maintained by all patients throughout the study period and tested for with multiple breath tests throughout the day. According to best clinical practice, a standardised TS regimen was advised to all patients: 5 days of intravenous 100 mg thiamine chloride hydrochloride in 100 mL 0.9% sodium chloride infusion three times per day, followed by 7 days of three times daily 100 mg orally administered thiamine administration, followed by 100 mg orally administered daily. In the case of any contraindication for intravenous administration or according to patient preference, an oral treatment regimen was applied with 7 days of three times daily 100 mg thiamine administration, followed by 100 mg daily. In both groups, the oral substitution with 100 mg thiamine per day was recommended as a permanent substitution beyond the duration of this study. Blood was collected and clinical parameters were assessed at baseline (i.e., Visit 1, prior to initiation of TS), at Visit 2 (Day 5–7), Visit 3 (Day 12–14) and at visit 4 (Day 54–56). In the current study, antibody titres were determined in serum at baseline and Visit 3. This study was approved by the Ethics committee of the Medical University of Vienna (EK‐No.: 1379/2023) and was conducted in accordance with the Declaration of Helsinki.

**Table:** 

	Included AUD cohort	
	*n* = 50	
Age (years)	47.9 (± 8.5)	
Sex (male)	27 (54%)	
Daily alcohol intake (gram)	154 (107–200)	
AUDIT score	32 (26.5–34)	
SESA score	60.9 (47–70.7)	
BDI score	20 (13.5–29.5)	
	Baseline (mean ± stdev)	14 days abstinence (mean ± stdev)
AST (U/L)	112.7 (±120.8)	35.3 (±26.4)
ALT (U/L)	66.1 (±52.4)	41.1 (±35.6)
GGT (U/L)	344.8 (±376.4)	176.5 (±186.4)
Bilirubin (mg/dL)	1.14 (±2.56)	0.53 (±0.72)

Abbreviations: AUDIT, alcohol use disorder identification test; BDI, Beck Depression Inventory—II; SESA, severity scale of alcohol dependence.

### 4.3 Antigen‐Specific Immunoglobulin Assessment in Serum of Humans

Chemiluminescent ELISA was performed as previously described [23]. Briefly, purified anti‐human IgM (555780, BD Pharmingen, San Jose, CA, USA) and IgG (I3382, Sigma‐Aldrich, Austria) at concentrations of 2 μg/mL, and antigens (MDA‐BSA, MAA‐BSA) at concentrations of 5 μg/mL in 50 μL phosphate‐buffered saline (PBS)‐EDTA were added to each well of a 96‐well round‐bottom microtitration plate and incubated overnight at 4°C. After washing and blocking with Tris‐buffered saline (TBS) with EDTA (pH 7.4, containing 1% bovine serum albumin (BSA), 30 min at room temperature), the plate was incubated with plasma samples in their respective dilutions (MDA‐BSA, MAA‐BSA IgM/IgG: 1:200) in 1% BSA in TBS with EDTA (pH 7.4) for 2 h at room temperature or overnight at 4°C. Alkaline phosphatase (AP)‐labelled goat anti‐human IgM (μ‐chain specific; A3437, Sigma‐Aldrich, Vienna, Austria; 1:30 000 in TBS‐BSA) or AP‐labelled goat anti‐human IgG (γ‐chain specific; A3178, Sigma‐Aldrich, Vienna, Austria; 1:50000 in TBS‐BSA) was used for detection. AP‐conjugated secondary reagents were detected using Lumi‐Phos (Lumigen, Southfield, Michigan, USA; 33% solution in water) and a Synergy 2 Luminometer (BioTek, Winooski, Vermont, USA). Washing steps were performed on an ELx405 Select Deep Well Microplate Washer (BioTek, Winooski, Vermont, USA) with PBS or PBS‐EDTA. Internal controls were included on each microtiter plate to detect potential variations between microtiter plates. The intra‐assay coefficient of variation for all assays was 5%–15%.

### 4.4 Animal Experiments

*sIgM* ^*−/−*^, *Siglec‐G* ^*−/−*^ and *C3* ^*−/−*^ mice were provided by Prof. CJ Binder (MUW, Vienna, Austria). All mice were on a C57BL/6J background. Age‐matched littermate controls aged 9 to 18 weeks were used in the studies. For all studies, female mice were used except when indicated differently. Mice were bred under specific pathogen‐free conditions at the Department of Biomedical Research or Department of Laboratory Animal Science and Genetics of the Medical University of Vienna, Austria. In the antibody administration experiment, mice were randomised into experimental groups and received 200 μg of LR04 (kind gift of Prof. Dr. J. L. Witztum, Univ. of California San Diego) or isotype control IgM administered intravenously via the tail vein on Day 5 and Day 11 of the diet intervention study. Female wildtype or *C3* ^*−/−*^ mice received γ‐irradiation (2 × 6 Gy) and were reconstituted with bone marrow transplant (4 × 10^6^ cells) isolated from *Siglec‐G* ^*−/−*^ and Wt littermates whose sex was matched to the recipient mice. Recipient mice had a 6‐week recovery period before further interventions to allow for haematopoietic compartment reconstitution. The order of mice and cages for specific treatments to mice was random. All experimental studies and interventions were approved by the Animal Ethics Committee of the Medical University of Vienna and the Austrian Federal Ministry of Education, Science and Research, and were performed according to Good Scientific Practice guidelines (Licence number: 335/19).

### 4.5 Dietary Interventions

For chronic‐binge ethanol diet model (NIAAA [49]), mice were fed with Lieber–DeCarli diet for 15 days. At Day 16, mice were gavaged with one dose of ethanol (5 g/kg BW) and sacrificed 8 h later as described. Liquid diet was freshly prepared three times/week with irradiated diet and was administered *ad libitum*. Mice on isocaloric control diet were pair‐fed to ethanol‐fed mice. Sacrifice of experimental mice occurred randomly with alternating order of treatment/genotype groups to prevent confounding effects of time of harvest. For all further analysis, measurements were done in random order and in a blinded fashion.

### 4.6 Biochemical Analyses in Murine Models

Blood was collected in EDTA collection tubes (Greiner Bio‐One, Germany), and plasma was obtained by centrifugation at 2000× *g* for 10 min. Plasma levels of ALT were determined using Reflotron ALT strips on a Reflotron Plus (Roche). Plasma levels of AST were determined using Exdia Liver Test 11 V (S109448) strips on an Element DC/EXDIA Samsung PT10V (Lab Technologies, Vienna, AT). Hepatic triglyceride levels were measured according to the manufacturer's instructions using Liquid Reagents kit (GPO‐PAP Triglyceride Liquicolor kit, HUMAN Biochemica and Diagnostica mbH, Wiesbaden, Germany). Protein content was measured using the Pierce BCA Protein Assay Kit (Thermo Fisher Scientific, Waltham, MA, USA). Plasma ethanol levels were determined using the Ethanol assay kit (MAK076‐1KT, Sigma‐Aldrich, Vienna, Austria) according to the manufacturer's protocol. Plasma IgM titres were measured using antimouse IgM (AP500, Sigma‐Aldrich, Austria) for coating and AP‐labelled antimouse IgM (A9688, Sigma‐Aldrich, Austria) for detection as described before [22]. For determining IgG1 levels in plasma, anti‐mouse IgG1 (Clone: RMG1‐1; 406602, BioLegend, San Diego, USA) was used for coating, and biotinylated antimouse IgG1 (Clone: A85‐1; 553441, Becton Dickinson, Austria) for detection. Total IgA antibody titres in plasma are measured by a chemiluminescent‐based sandwich ELISA using antimouse IgA (Clone C10‐3; 556969, Becton Dickinson, Austria) for coating and biotinylated antimouse IgA (Clone C10‐1; 556978, Becton Dickinson, Austria) for detection. MDA, MAA and PC specific antibodies were assessed by coating with 5 μg/mL of MDA‐BSA, MAA‐BSA or PC‐BSA and using AP‐labelled antimouse IgM (A9688, Sigma‐Aldrich, Austria) or biotinylated antimouse IgG1 (Clone: A85‐1; 553441, Becton Dickinson, Austria) for detection.

### 4.7 Immunohistochemistry

Mouse liver sections were embedded in OCT compound, and 7 μm frozen sections were stained and quantified for CD11b (Mac‐1; Clone: M1/70, 550282, Becton Dickinson, Austria) for infiltrating macrophages and neutrophils, for C3b deposition (C3b/iC3b, C3c; Clone: 3/26; HM1078, Hycult Biotech, Uden, Netherlands) and with Oil Red O (Sigma‐Aldrich, Vienna, Austria) to determine lipid content as done previously [20]. At least five high‐power fields (100× or 200× magnification) of the liver per mouse were randomly selected for quantification of positive cells per field.

Formalin‐fixed liver samples were embedded in paraffin and 4 μm sections were stained for Mayer's Haematoxylin solution (254766, AppliChem, Darmstadt, Germany) and Eosin Y solution (HT110321, Sigma‐Aldrich, Vienna, Austria) for assessing general tissue morphology and with Sirius Red (365548, Sigma‐Aldrich, Vienna, Austria) for the assessment of liver fibrosis, which was scored for collagen deposition in a blinded manner by an experienced hepatologist as described previously [50]. Moreover, liver tissue sections were stained for IgM levels in the liver using antimouse IgM (AP500, Sigma‐Aldrich, Austria) and HRP‐labelled polyclonal antigoat IgG antibody (81‐1620, Invitrogen/Thermo Fisher Scientific, Waltham, MA, USA). Staining for F4/80 positive cells (F4/80 antibody, Abcam Cambridge, UK) and Ly6G/Ly6C‐positive cells (NIMP‐R14, Thermofisher, MA1‐40038) was done as described previously [20, 51, 52]. Positive cells were counted in six to eight microscopic fields (magnification 200×) of each liver section to determine means. For staining of cleaved caspase‐3 and αSMA, sections were incubated after endogenous peroxidase blocking with the respective primary antibodies (cleaved caspase‐3 (Asp175) (5A1E), Cell Signalling Technology, Danvers, MA, USA; αSMA: EPR 5368, Abcam Cambridge, UK) overnight at 4°C. Subsequently, sections were incubated with peroxidase‐linked secondary antibodies followed by diaminobenzidine (Peroxidase Envision Kit, DAKO, Hamburg, Germany). Staining was evaluated using a camera integrated in a microscope (Leica DM4000 B LED, Leica, Wetzlar, Germany) and an analysis system (Leica Applications Suite, Leica, Wetzlar, Germany) as previously described [51]. Data from eight fields (magnification 200×) of each tissue section were used to calculate means of areas positively stained.

### 4.8 Flow Cytometry

Single‐cell suspensions for flow cytometric analysis of the liver were prepared as described previously by us [50, 53]. Blocking and staining of single cells were performed in 2% heat‐inactivated FBS in PBS at 4°C in the dark. For blocking unspecific Fc receptor interactions, cells were incubated with unconjugated anti‐CD16/CD32 antibody (clone 93; 14‐0161‐85, eBioscience, Invitrogen). After washing, cells were incubated with the following antibodies for 20 min: anti‐CD45 PerCP‐Cy5.5 (clone: 30‐F11; 45‐0451‐82, eBioscience, Invitrogen) or anti‐CD45 FITC (clone: 30F‐11; 103 108, BioLegend), anti‐B220 AF700 (clone: RA3‐6B2; B103231, BioLegend), anti‐CD3 PE (clone: 145‐2C11; 12‐0031‐82, eBioscience, Invitrogen), anti‐CD4 FITC (clone: GK1.5; 11‐0041‐82, eBioscience, Invitrogen), anti‐CD8 APC (clone: 53‐6.7; 17‐0081‐82, eBioscience, Invitrogen), anti‐F4/80 PE‐Cy7 (clone: BM8; 123 113, BioLegend), anti‐CD11b AF700 (clone: M1/70; 56‐0112‐82, eBioscience, Invitrogen), anti‐Ly6G PE (clone: 1A8; 127 608, Biolegend), anti‐CD11c APC‐Cy7 (clone: N418; 47‐0114‐82, eBioscience, Invitrogen), anti‐NK1.1 PerCP‐Cy5.5 (clone: PK136; 108 727, BioLegend). Samples were stained and acquired on a BD Fortessa. Analysis was performed using FlowJo Software V10 and cell populations were identified as shown according to the gating strategy in Figure S10 (FlowJo Inc., BD Life Sciences).

### 4.9 RNA Isolation

For whole liver RNA isolation, 50 mg tissue pieces from the left lateral liver lobes were snap‐frozen in liquid nitrogen. Frozen tissue in QIAzol lysis reagent was homogenised mechanically using stainless steel beads in Tissue Lyser II (Qiagen, Hilden, Germany). RNA was extracted using QIAzol according to the manufacturer's instructions. RNA content and quality were assessed using a Nanodrop spectrophotometer (PeqLab).

### 4.10 cDNA Generation and qPCR

For quantitative real‐time PCR, up to 1 μg of RNA was reverse transcribed using the High‐Capacity cDNA Reverse Transcription kit (Applied Biosystems, Thermo Fisher, Waltham, MA, USA) to generate cDNA. RT PCR was performed on a CFX96 Real‐Time PCR System (Bio‐Rad Laboratories, Hercules, CA, USA) using the KAPA SYBR FAST kit (Thermo Fisher, Waltham, MA, USA) and the primers listed below. Gene expression was normalised to *18S* or *Cyclophilin B* where indicated.

**Table:** 

Gene	Forward sequence 5′‐3′	Reverse sequence 5′‐3′
*18S*	AGTCCCTGCCCTTTGTACACA	CGATCCCAGGGCCTCACTA
*Adh1*	GGGTTCTCAACTGGCTATGG	ACAGACAGACCGACACCTCC
*C3*	CTGACTCTGTGTGGGTGGAT	AGCCAATGTCTGCCTTCTCT
*Ccr2*	CAGGTGACAGAGACTCTTGGAATG	GAACTTCTCTCCAACAAAGGCATAA
*Cd11b*	ATGGACGCTGATGGCAATACC	TCCCCATTCACGTCTCCCA
*Col1a1*	AACCCTGCCCGCACATG	CAGACGGCTGAGTAGGGAACA
*Col3a1*	TACACCTGCTCCTGTGCTTC	CATTCCTCCCACTCCAGACT
*Cxcl1*	GCTGGGATTCACCTCAAGAA	TCTCCGTTACTTGGGGACAC
*Cyp2e1*	CTTAGGGAAAACCTCCGCAC	GGGACATTCCTGTGTTCCAG
*Cyclophilin B*	CAGCAAGTTCCATCGTGTCATCAAGG	GGAAGCGCTCACCATAGATGCTC
*Il1β*	TGTGCAAGTGTCTGAAGCAGC	TGGAAGCAGCCCTTCATCTT
*Il10*	GCCTTATCGGAAATGATCCA	TTTTCACAGGGGAGAAATCG
*Ly6g*	CATTGCAAAGTCCTGTGTGC	AGGGGCAGGTAGTTGTGTTG
*Tgfβ*	GTCCTTGCCCTCTACAACCA	GTTGGACAACTGCTCCACCT

### 4.11 Statistical Analysis

Data comparing two groups were assessed as appropriate by two‐tailed unpaired Student's *t*‐test following the evaluation of Gaussian distribution. Multiple datasets were compared using one‐way analysis of variance (ANOVA) with Bonferroni correction. After normality testing using the Shapiro–Wilk test, Pearson's or Spearman's (for non‐normally distributed data) correlation coefficient was determined to assess potential correlations between human blood markers. Statistical analyses were performed using GraphPad Prism v10. The results are expressed as mean ± standard error (SEM) unless stated otherwise. Statistical significance was set at *p* ≤ 0.05.

## Author Contributions

D.R., B.P.S., T.B., T.D. and C.H. performed part of the studies, acquired and analysed the data, and critically revised the manuscript. N.P.‐M., C.J.B. provided technical assistance, reagents and critically revised the manuscript. K.B. and I.B. provided technical assistance, reagents, acquired and analysed part of the data and critically revised the manuscript. B.S., B.S.H., M.M., and T.R. provided the human ALD samples, acquired part of the data, analysed the data and edited the manuscript. S.L., S.W., B.V. and D.K. provided the human AUD samples, acquired and analysed part of the data and edited the manuscript. T.H. designed and performed the studies, acquired and analysed the data and wrote and edited the manuscript.

## Conflicts of Interest

B.S. received travel support from AbbVie, Gilead and Falk. B.S.H. received travel support from Ipsen. M.M. served as a speaker and/or consultant and/or advisory board member for AbbVie, Gilead, Collective Acumen and W. L. Gore & Associates and received travel support from AbbVie and Gilead. T.R. received grant support from Abbvie, Boehringer Ingelheim, Gilead, Intercept/Advanz Pharma, MSD, Myr Pharmaceuticals, Philips Healthcare, Pliant, Siemens and W. L. Gore & Associates; speaking honoraria from Abbvie, Echosens, Gilead, Intercept/Advanz Pharma, Roche, MSD, W. L. Gore & Associates; consulting/advisory board fees from Abbvie, Astra Zeneca, Bayer, Boehringer Ingelheim, Gilead, Intercept/Advanz Pharma, MSD, Resolution Therapeutics, Siemens; and travel support from Abbvie, Boehringer Ingelheim, Dr. Falk Pharma, Gilead and Roche. All other authors declare no conflicts of interest.

## Supporting information


**Figure S1:** Systemic total and anti‐MDA IgM and IgG antibody titres during human ALD. (A) Serum total IgM levels in patients with ALD, classified according to the CHILD score. (B) Serum total IgM levels in patients with ALD, classified according to the MELD score. (C) Serum total IgG levels in patients with ALD, classified according to the CHILD score. (D) Serum total IgG levels in patients with ALD, classified according to the MELD score. (E) Serum anti‐MDA IgM levels in patients with ALD, classified according to the CHILD score. (F) Serum anti‐MDA IgM levels in patients with ALD, classified according to the MELD score. (G) Serum anti‐MDA IgG levels in patients with ALD, classified according to the CHILD score. (H) Serum anti‐MDA IgG levels in patients with ALD, classified according to the MELD score. (I) Correlation analyses between serum AST (IU/L) levels and anti‐MDA IgM and IgG titres in all ALD patients, stratified into compensated or decompensated cirrhosis. (J) Correlation analyses between HVPG (mmHg) measurement and anti‐MDA IgM and IgG titres in all ALD patients, stratified into compensated or decompensated cirrhosis. (K) Correlation analyses between serum CRP levels and antibody titres in patients with ALD. (L) Correlation analyses between serum IL6 levels and antibody titres in patients with ALD. Data shown as mean ± SEM of *n* = 204 patients. *r* indicates Spearman's correlation coefficient. *p ≤ 0.05, **p ≤ 0.01, ***p ≤ 0.001.

**Figure S2:** Murine ALD development in *sIgM*‐deficient mice after chronic‐binge ethanol feeding. (A) Liver‐to‐body weight ratio. (B) Hepatic CD8^+^ T cells as percentage of CD45^+^ cells assessed by flow cytometry. (C) Hepatic CD4^+^ T cells as percentage of CD45^+^ cells assessed by flow cytometry. (D) Hepatic natural killer cells (NK1.1^+^) as percentage of CD45^+^ cells assessed by flow cytometry. (E) Hepatic dendritic cells (CD11C^+^) as percentage of CD45^+^ cells assessed by flow cytometry. (F–I) mRNA levels of indicated genes (*Il10*, *Tgfβ*, *Col1a1*, *Col3a1*) in livers of ethanol‐fed mice, assessed by qPCR. Data are shown relative to wildtype mice and normalised to *Cyclophilin B*. (J) Plasma ethanol levels at the study endpoint. (K, L) mRNA levels of indicated genes (*Cyp2e1*, *Adh1*) in livers of ethanol‐fed mice, assessed by qPCR. Data are shown relative to the respective wildtype mice and normalised to *18S*. (L) Plasma IgA levels. (M) Plasma IgG1 levels. (N) Plasma anti‐MDA IgG1 levels. Data shown as mean ± SEM of *n* = 10–17/group (A, F–O) or *n* = 5–10/group (B–E). *p ≤ 0.05, **p ≤ 0.01, ***p ≤ 0.001, ****p ≤ 0.0001.

**Figure S3:** Plasma anti‐MDA and anti‐MAA IgM levels in wildtype mice after chronic‐binge ethanol or isocaloric control diet feeding. (A) Plasma anti‐MDA IgM titers in wildtype mice after chronic‐binge ethanol diet or isocaloric control diet, normalised to total IgM levels. A.U.: Arbitrary Units. (B) Plasma anti‐MAA IgM titers in wildtype mice after chronic‐binge ethanol diet or isocaloric control diet, normalised to total IgM levels. A.U., arbitrary units. Data shown as mean ± SEM of *n* = 14/group. **p ≤ 0.01.

**Figure S4:** Plasma antibody titres in *Siglec‐G* ^*−/−*^ and wildtype mice after isocaloric control diet feeding. (A–C) Plasma total IgM (A), anti‐MAA IgM (B) and anti‐MDA IgM (C) levels in *Siglec‐G* ^*−/−*^ mice and Wt mice fed an isocaloric control diet. (D–F) Plasma total IgG1 (D), anti‐MAA IgG1 (E) and anti‐MDA IgG1 (F) levels in *Siglec‐G* ^*−/−*^ mice and Wt mice fed an isocaloric control diet. (G) Plasma IgA levels in *Siglec‐G* ^*−/−*^ mice and Wt mice fed an isocaloric control diet. Data shown as mean ± SEM of *n* = 6–5 mice/group. *p ≤ 0.05, **p ≤ 0.01.

**Figure S5:** Profiling *Siglec‐G* ^*−/−*^ and wildtype mice after chronic‐binge ethanol feeding. (A) Representative images of IGM deposition in the liver of *Siglec‐G* ^*−/−*^ mice and Wt mice after ethanol diet as depicted in Figure 4A. (B) Plasma IgA levels in *Siglec‐G* ^*−/−*^ mice and Wt mice‐fed ethanol diet. (C) Plasma IgG1 levels in *Siglec‐G* ^*−/−*^ mice and Wt mice‐fed ethanol diet. (D) Plasma anti‐MDA IgG1 titres in *Siglec‐G* ^*−/−*^ mice and Wt mice‐fed ethanol diet. (E) Liver‐to‐body weight ratio. (F) Hepatic CD8^+^ T cells as percentage of CD45^+^ cells assessed by flow cytometry. (G) Hepatic CD4^+^ T cells as percentage of CD45^+^ cells assessed by flow cytometry. (F) Hepatic natural killer cells (NK1.1^+^) as percentage of CD45^+^ cells assessed by flow cytometry. (G) Hepatic dendritic cells (CD11C^+^) as percentage of CD45^+^ cells assessed by flow cytometry. (J, K) mRNA levels of indicated genes (*Tgfβ, Il10*) in livers of ethanol‐fed *Siglec‐G* ^*−/−*^ and Wt mice, assessed by qPCR. Data are shown relative to wildtype mice and normalised to *Cyclophilin B*. (L) Plasma ethanol levels. (M, N) mRNA levels of indicated genes (*Cyp2e1*, *Adh1*) in livers of ethanol‐fed *Siglec‐G* ^*−/−*^ and Wt mice, assessed by qPCR. Data are shown relative to the respective wildtype mice and normalised to *18S*. Data shown as mean ± SEM of *n* = 17–16/group (B–E, J–N) or *n* = 6–10/group (F–I).

**Figure S6:** Profiling of wildtype mice treated with LR04 or isotype control antibodies in the chronic‐binge ethanol feeding model. (A) Plasma IgA levels in LR04 and isotype control‐treated wildtype mice after chronic‐binge ethanol diet as shown in Figure 5A. (B) Plasma anti‐PC IgM levels. (C) Plasma IgG1 levels. (D) Plasma anti‐MAA IgG1 levels. (E) Plasma anti‐MDA IgG1 levels. (F) Liver‐to‐body weight ratio. (G–J) mRNA levels of indicated genes (*Ly6g*, *Il10*, *Tgfβ*, *Col3a1*) in livers of ethanol‐fed LR04 and isotype control‐treated wildtype mice, assessed by qPCR. Data are shown relative to wildtype mice and normalised to *Cyclophilin B*. (K) Plasma ethanol levels. (L, M) mRNA levels of indicated genes (*Cyp2e1*, *Adh1*) in livers of ethanol‐fed LR04 and isotype control‐treated wildtype mice, assessed by qPCR. Data are shown relative to the respective wildtype mice and normalised to *18S*. Data shown as mean ± SEM of *n* = 14–12 mice/group. *p ≤ 0.05.

**Figure S7:** Chronic‐binge ethanol diet in mice lacking C3 does not affect ALD development. (A) Schematic of chronic‐binge ethanol feeding study in female (white dots) and male (red dots) Wt (white) and *C3* ^*−/−*^ (black) mice. (B) Plasma IgM levels at the end of the study. (C) Plasma anti‐MAA and anti‐MDA IgM levels at the end of the study. (D) Plasma ALT levels. (E) Plasma AST levels. (F) Representative images showing H&E staining of liver sections. Scale bars indicate 100 μm. (G) Liver‐to‐body weight ratio. (H) Hepatic triglyceride content. (I) Flow cytometry analysis of neutrophils (Ly6G^+^), monocytes (CD11B^+^F4/80^−^), and Kupffer cells (CD11B^+/−^F4/80^+^) in the liver. Data are shown relative to the total amount of immune cells present (CD45^+^). (J–M) mRNA levels of indicated genes (*Cxcl1*, *Ccr2, Il1β*, *Tgfβ*) in livers of ethanol‐fed mice Wt and *C3* ^*−/−*^ mice, assessed by qPCR. Data are shown relative to wildtype mice and normalised to *Cyclophilin B*. (N) Plasma ethanol levels. (O, P) mRNA levels of indicated genes (*Cyp2e1*, *Adh1*) in livers of ethanol‐fed mice Wt and *C3* ^*−/−*^ mice, assessed by qPCR. Data are shown relative to wildtype mice and normalised to *18S*. Data shown as mean ± SEM of *n* = 10/group.

**Figure S8:** Profiling of ethanol‐fed *C3* ^*−/−*^ and Wt mice after transplantation with *Siglec‐G* ^*−/−*^ bone marrow. (A) Plasma IgG1 levels at the end of the study depicted in Figure 7A. (B) Plasma anti‐MDA IgG1 levels at the end of the study. (C–H) mRNA levels of indicated genes (*C3*, *Il10, Tgfβ*, *Col3a1*, *Cyp2e1*, *Adh1*) in livers, assessed by qPCR. Data are shown relative to wildtype mice and normalised to *Cyclophilin B*. Data shown as mean ± SEM of *n* = 8/6/group. ****p ≤ 0.0001.

**Figure S9:** Systemic anti‐MDA IgM correlate with altered complement factors in ALD patients. (A) Correlation analyses between serum C3c (mg/dL) and anti‐MDA IgM and IgG titres in ALD patients, stratified into compensated or decompensated cirrhosis. (B) Correlation analyses between serum C4 (mg/dL) and anti‐MDA IgM and IgG titres in ALD patients, stratified into compensated or decompensated cirrhosis. Data shown as mean ± SEM of *n* = 204 patients. *r* indicates Spearman's correlation coefficient. ****p ≤ 0.0001.

**Figure S10:** Flow cytometry gating strategies. (A) Representative images of the gating strategy applied to identify hepatic neutrophils, natural killer cells, dendritic cells, monocytes, and Kupffer cells by flow cytometry. (B) Representative images of the gating strategy applied to identify hepatic B cells, T cells, CD4 T helper cells and CD8 cytotoxic T cells by flow cytometry.

**Table S1:** Correlation analyses of serum markers in human ALD.

**Table S2:** Correlation analyses of serum markers in human AUD.

## Data Availability

Data are available upon reasonable request to the corresponding author.
