# Supplementary figures and images for: IgM Antibodies Targeting Malondialdehyde Promote Complement‐Mediated Liver Injury in Alcohol‐Related Liver Disease
Source: Liver Int. 2025 Sep 17;45(10):e70356. doi: 10.1111/liv.70356 (PMC12442528; doi:10.1111/liv.70356)

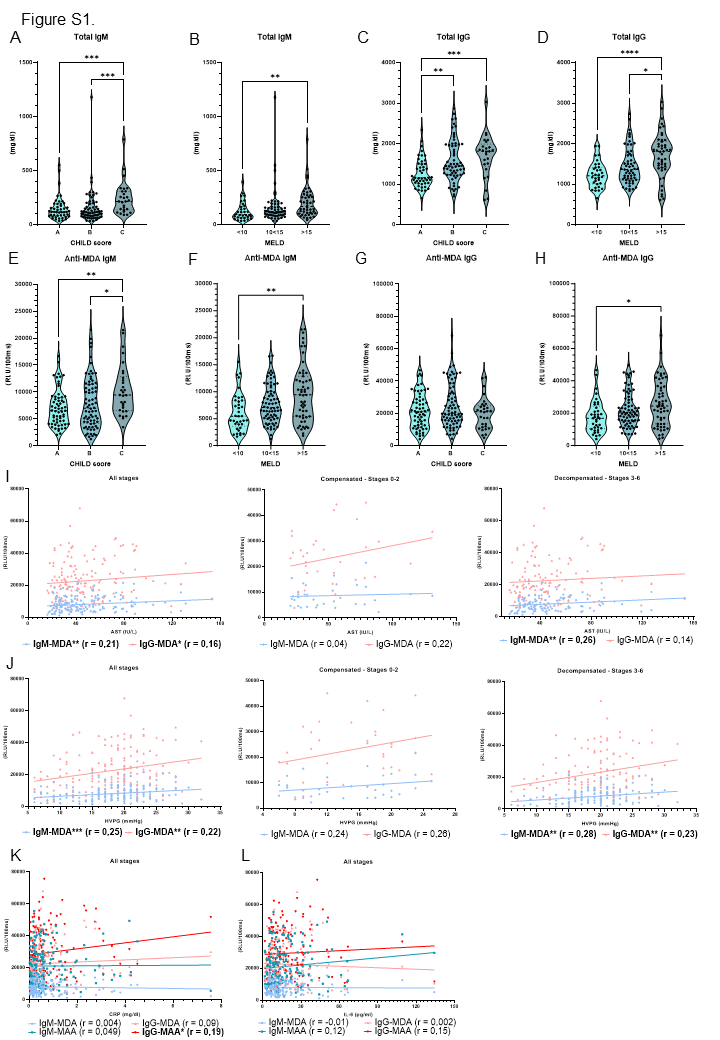

Supplement: Supplementary file 1 — Figure S1: Systemic total and anti‐MDA IgM and IgG antibody titres during human ALD. (A) Serum total IgM levels in patients with ALD, classified according to the CHILD score. (B) Serum total IgM levels in patients with ALD, classified according to the MELD score. (C) Serum total IgG levels in patients with ALD, classified according to the CHILD score. (D) Serum total IgG levels in patients with ALD, classified according to the MELD score. (E) Serum anti‐MDA IgM levels in patients with ALD, classified according to the CHILD score. (F) Serum anti‐MDA IgM levels in patients with ALD, classified according to the MELD score. (G) Serum anti‐MDA IgG levels in patients with ALD, classified according to the CHILD score. (H) Serum anti‐MDA IgG levels in patients with ALD, classified according to the MELD score. (I) Correlation analyses between serum AST (IU/L) levels and anti‐MDA IgM and IgG titres in all ALD patients, stratified into compensated or decompensated cirrhosis. (J) Correlation analyses between HVPG (mmHg) measurement and anti‐MDA IgM and IgG titres in all ALD patients, stratified into compensated or decompensated cirrhosis. (K) Correlation analyses between serum CRP levels and antibody titres in patients with ALD. (L) Correlation analyses between serum IL6 levels and antibody titres in patients with ALD. Data shown as mean ± SEM of n = 204 patients. r indicates Spearman's correlation coefficient. *p ≤ 0.05, **p ≤ 0.01, ***p ≤ 0.001. [file LIV-45-0-s004.png]

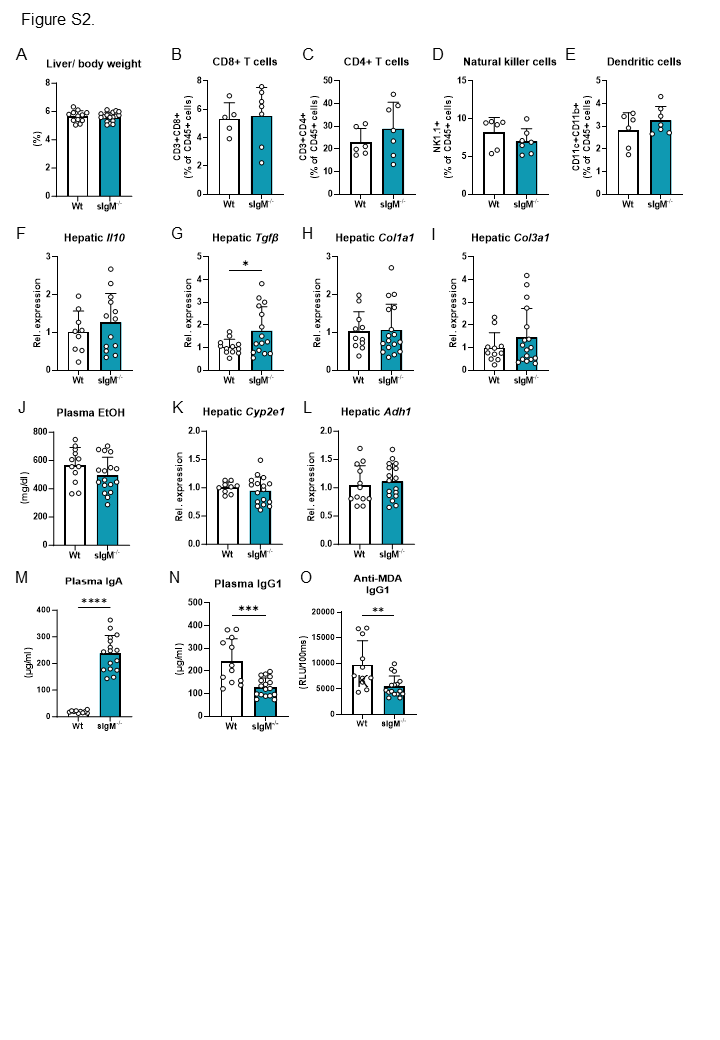

Supplement: Supplementary file 2 — Figure S2: Murine ALD development in sIgM‐deficient mice after chronic‐binge ethanol feeding. (A) Liver‐to‐body weight ratio. (B) Hepatic CD8+ T cells as percentage of CD45+ cells assessed by flow cytometry. (C) Hepatic CD4+ T cells as percentage of CD45+ cells assessed by flow cytometry. (D) Hepatic natural killer cells (NK1.1+) as percentage of CD45+ cells assessed by flow cytometry. (E) Hepatic dendritic cells (CD11C+) as percentage of CD45+ cells assessed by flow cytometry. (F–I) mRNA levels of indicated genes (Il10, Tgfβ, Col1a1, Col3a1) in livers of ethanol‐fed mice, assessed by qPCR. Data are shown relative to wildtype mice and normalised to Cyclophilin B. (J) Plasma ethanol levels at the study endpoint. (K, L) mRNA levels of indicated genes (Cyp2e1, Adh1) in livers of ethanol‐fed mice, assessed by qPCR. Data are shown relative to the respective wildtype mice and normalised to 18S. (L) Plasma IgA levels. (M) Plasma IgG1 levels. (N) Plasma anti‐MDA IgG1 levels. Data shown as mean ± SEM of n = 10–17/group (A, F–O) or n = 5–10/group (B–E). *p ≤ 0.05, **p ≤ 0.01, ***p ≤ 0.001, ****p ≤ 0.0001. [file LIV-45-0-s009.png]

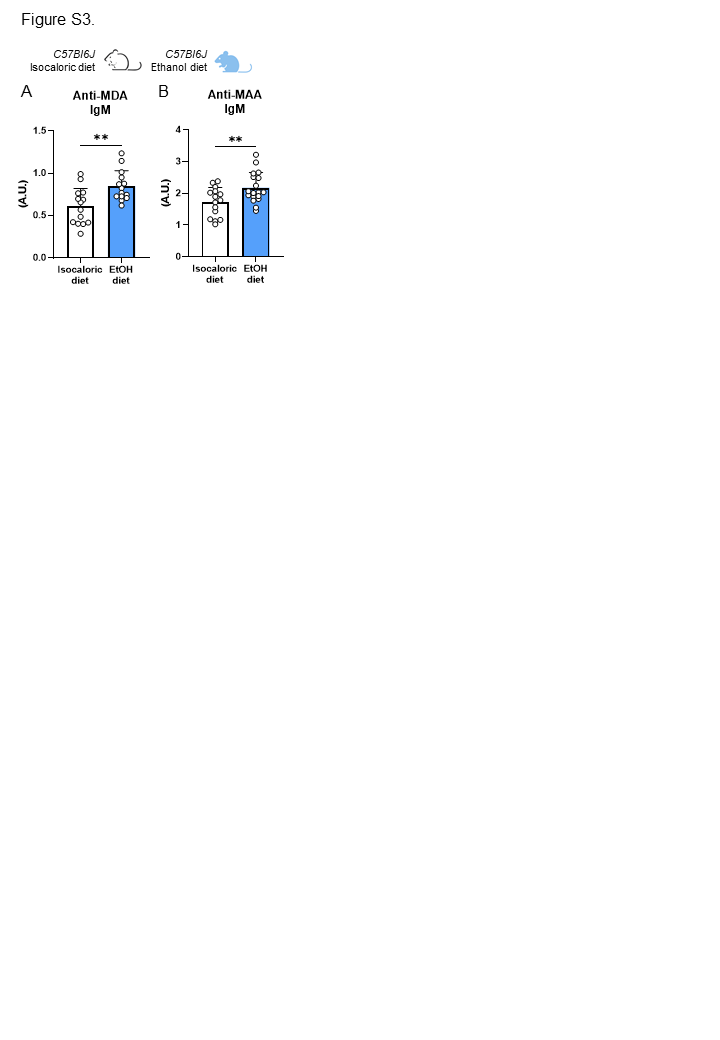

Supplement: Supplementary file 3 — Figure S3: Plasma anti‐MDA and anti‐MAA IgM levels in wildtype mice after chronic‐binge ethanol or isocaloric control diet feeding. (A) Plasma anti‐MDA IgM titers in wildtype mice after chronic‐binge ethanol diet or isocaloric control diet, normalised to total IgM levels. A.U.: Arbitrary Units. (B) Plasma anti‐MAA IgM titers in wildtype mice after chronic‐binge ethanol diet or isocaloric control diet, normalised to total IgM levels. A.U., arbitrary units. Data shown as mean ± SEM of n = 14/group. **p ≤ 0.01. [file LIV-45-0-s012.png]

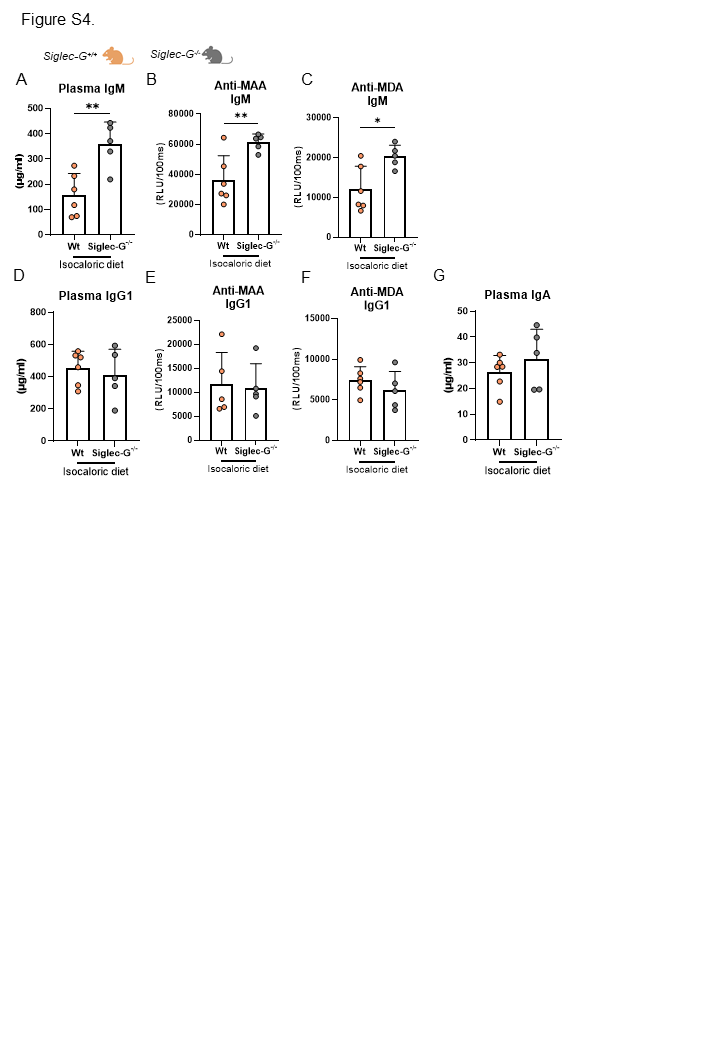

Supplement: Supplementary file 4 — Figure S4: Plasma antibody titres in Siglec‐G −/− and wildtype mice after isocaloric control diet feeding. (A–C) Plasma total IgM (A), anti‐MAA IgM (B) and anti‐MDA IgM (C) levels in Siglec‐G −/− mice and Wt mice fed an isocaloric control diet. (D–F) Plasma total IgG1 (D), anti‐MAA IgG1 (E) and anti‐MDA IgG1 (F) levels in Siglec‐G −/− mice and Wt mice fed an isocaloric control diet. (G) Plasma IgA levels in Siglec‐G −/− mice and Wt mice fed an isocaloric control diet. Data shown as mean ± SEM of n = 6–5 mice/group. *p ≤ 0.05, **p ≤ 0.01. [file LIV-45-0-s007.png]

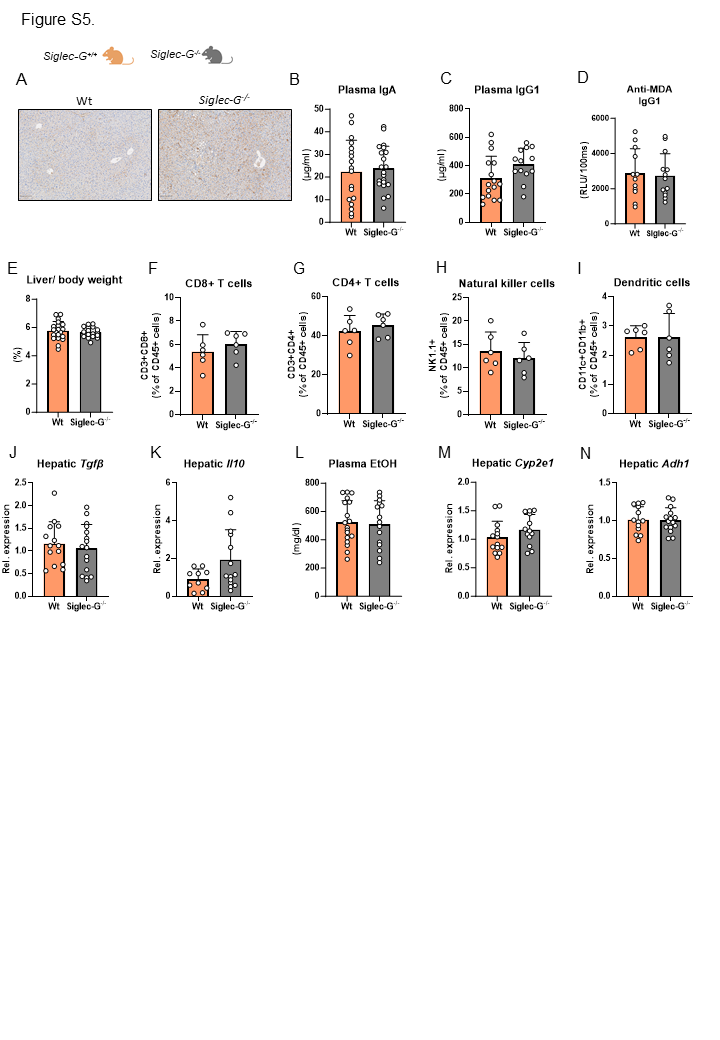

Supplement: Supplementary file 5 — Figure S5: Profiling Siglec‐G −/− and wildtype mice after chronic‐binge ethanol feeding. (A) Representative images of IGM deposition in the liver of Siglec‐G −/− mice and Wt mice after ethanol diet as depicted in Figure 4A. (B) Plasma IgA levels in Siglec‐G −/− mice and Wt mice‐fed ethanol diet. (C) Plasma IgG1 levels in Siglec‐G −/− mice and Wt mice‐fed ethanol diet. (D) Plasma anti‐MDA IgG1 titres in Siglec‐G −/− mice and Wt mice‐fed ethanol diet. (E) Liver‐to‐body weight ratio. (F) Hepatic CD8+ T cells as percentage of CD45+ cells assessed by flow cytometry. (G) Hepatic CD4+ T cells as percentage of CD45+ cells assessed by flow cytometry. (F) Hepatic natural killer cells (NK1.1+) as percentage of CD45+ cells assessed by flow cytometry. (G) Hepatic dendritic cells (CD11C+) as percentage of CD45+ cells assessed by flow cytometry. (J, K) mRNA levels of indicated genes (Tgfβ, Il10) in livers of ethanol‐fed Siglec‐G −/− and Wt mice, assessed by qPCR. Data are shown relative to wildtype mice and normalised to Cyclophilin B. (L) Plasma ethanol levels. (M, N) mRNA levels of indicated genes (Cyp2e1, Adh1) in livers of ethanol‐fed Siglec‐G −/− and Wt mice, assessed by qPCR. Data are shown relative to the respective wildtype mice and normalised to 18S. Data shown as mean ± SEM of n = 17–16/group (B–E, J–N) or n = 6–10/group (F–I). [file LIV-45-0-s002.png]

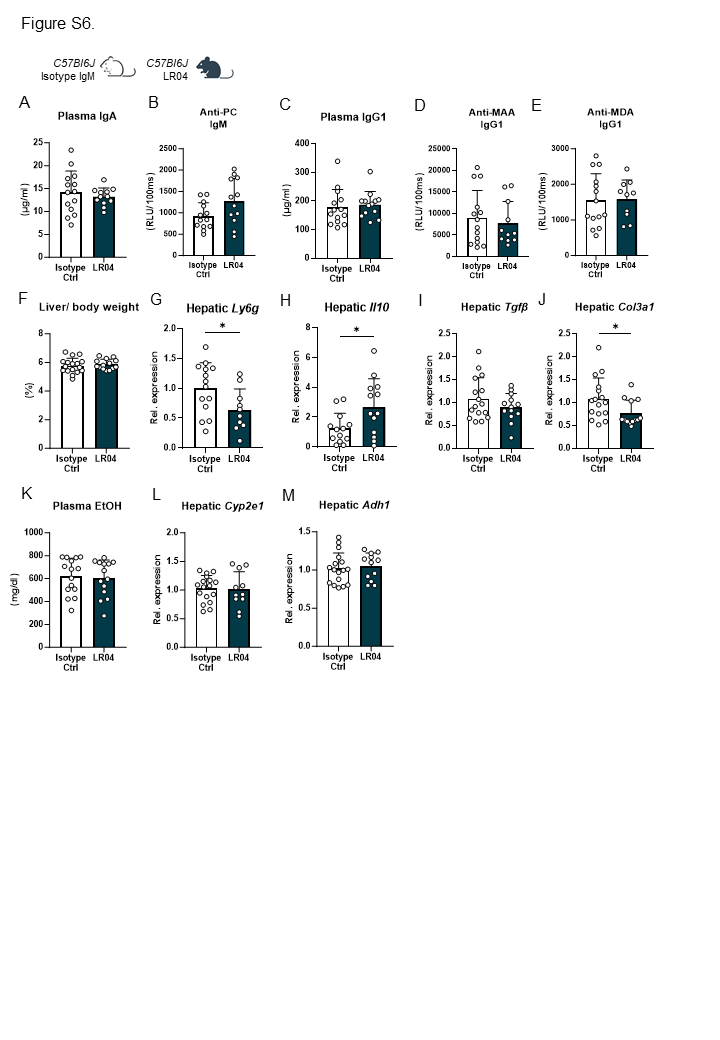

Supplement: Supplementary file 6 — Figure S6: Profiling of wildtype mice treated with LR04 or isotype control antibodies in the chronic‐binge ethanol feeding model. (A) Plasma IgA levels in LR04 and isotype control‐treated wildtype mice after chronic‐binge ethanol diet as shown in Figure 5A. (B) Plasma anti‐PC IgM levels. (C) Plasma IgG1 levels. (D) Plasma anti‐MAA IgG1 levels. (E) Plasma anti‐MDA IgG1 levels. (F) Liver‐to‐body weight ratio. (G–J) mRNA levels of indicated genes (Ly6g, Il10, Tgfβ, Col3a1) in livers of ethanol‐fed LR04 and isotype control‐treated wildtype mice, assessed by qPCR. Data are shown relative to wildtype mice and normalised to Cyclophilin B. (K) Plasma ethanol levels. (L, M) mRNA levels of indicated genes (Cyp2e1, Adh1) in livers of ethanol‐fed LR04 and isotype control‐treated wildtype mice, assessed by qPCR. Data are shown relative to the respective wildtype mice and normalised to 18S. Data shown as mean ± SEM of n = 14–12 mice/group. *p ≤ 0.05. [file LIV-45-0-s001.png]

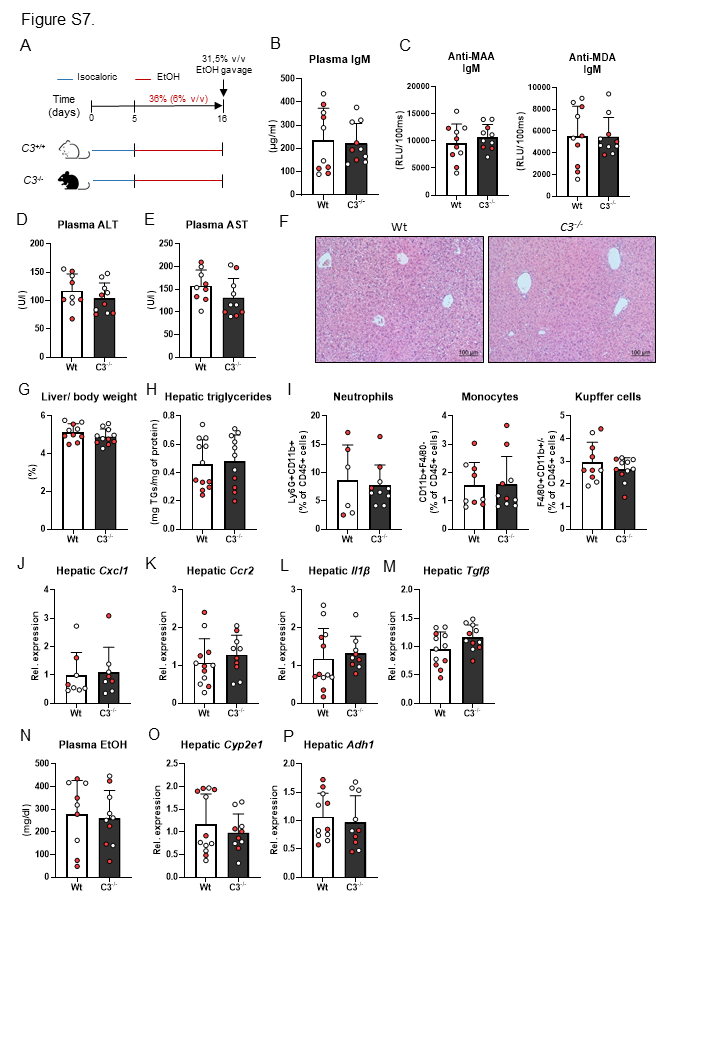

Supplement: Supplementary file 7 — Figure S7: Chronic‐binge ethanol diet in mice lacking C3 does not affect ALD development. (A) Schematic of chronic‐binge ethanol feeding study in female (white dots) and male (red dots) Wt (white) and C3 −/− (black) mice. (B) Plasma IgM levels at the end of the study. (C) Plasma anti‐MAA and anti‐MDA IgM levels at the end of the study. (D) Plasma ALT levels. (E) Plasma AST levels. (F) Representative images showing H&E staining of liver sections. Scale bars indicate 100 μm. (G) Liver‐to‐body weight ratio. (H) Hepatic triglyceride content. (I) Flow cytometry analysis of neutrophils (Ly6G+), monocytes (CD11B+F4/80−), and Kupffer cells (CD11B+/−F4/80+) in the liver. Data are shown relative to the total amount of immune cells present (CD45+). (J–M) mRNA levels of indicated genes (Cxcl1, Ccr2, Il1β, Tgfβ) in livers of ethanol‐fed mice Wt and C3 −/− mice, assessed by qPCR. Data are shown relative to wildtype mice and normalised to Cyclophilin B. (N) Plasma ethanol levels. (O, P) mRNA levels of indicated genes (Cyp2e1, Adh1) in livers of ethanol‐fed mice Wt and C3 −/− mice, assessed by qPCR. Data are shown relative to wildtype mice and normalised to 18S. Data shown as mean ± SEM of n = 10/group. [file LIV-45-0-s006.png]

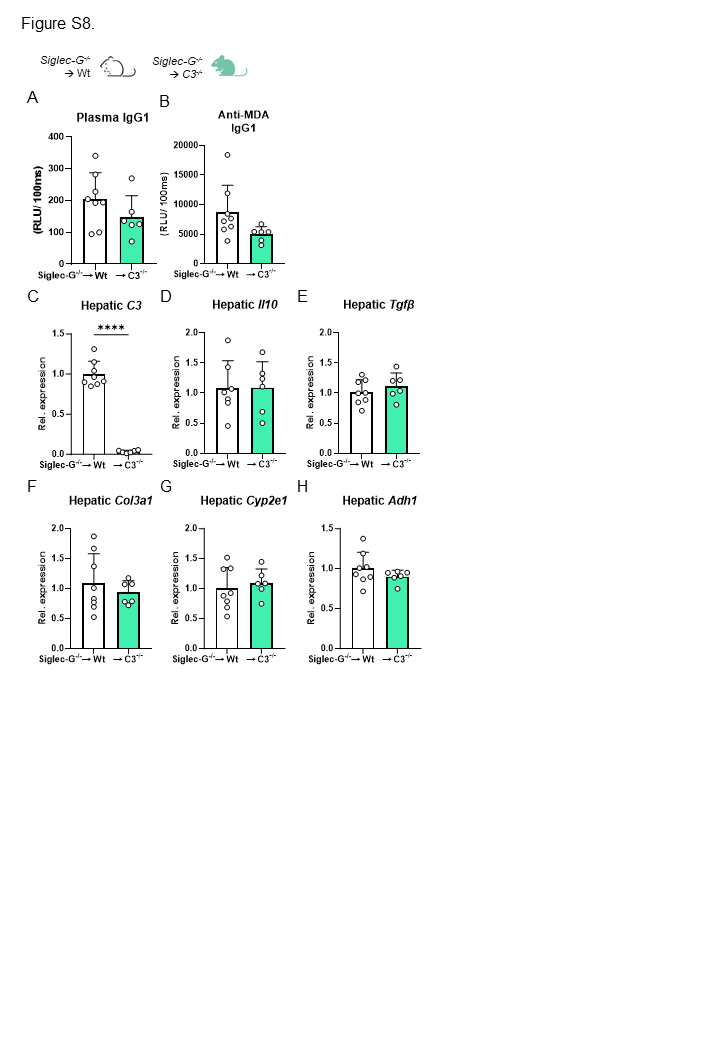

Supplement: Supplementary file 8 — Figure S8: Profiling of ethanol‐fed C3 −/− and Wt mice after transplantation with Siglec‐G −/− bone marrow. (A) Plasma IgG1 levels at the end of the study depicted in Figure 7A. (B) Plasma anti‐MDA IgG1 levels at the end of the study. (C–H) mRNA levels of indicated genes (C3, Il10, Tgfβ, Col3a1, Cyp2e1, Adh1) in livers, assessed by qPCR. Data are shown relative to wildtype mice and normalised to Cyclophilin B. Data shown as mean ± SEM of n = 8/6/group. ****p ≤ 0.0001. [file LIV-45-0-s011.png]

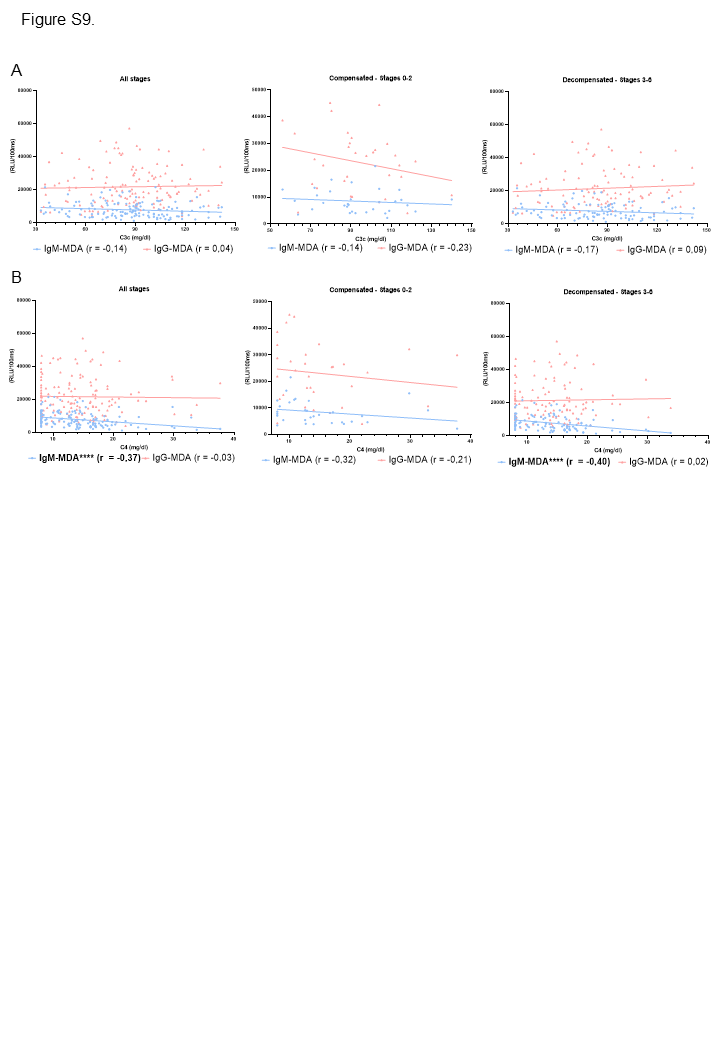

Supplement: Supplementary file 9 — Figure S9: Systemic anti‐MDA IgM correlate with altered complement factors in ALD patients. (A) Correlation analyses between serum C3c (mg/dL) and anti‐MDA IgM and IgG titres in ALD patients, stratified into compensated or decompensated cirrhosis. (B) Correlation analyses between serum C4 (mg/dL) and anti‐MDA IgM and IgG titres in ALD patients, stratified into compensated or decompensated cirrhosis. Data shown as mean ± SEM of n = 204 patients. r indicates Spearman's correlation coefficient. ****p ≤ 0.0001. [file LIV-45-0-s005.png]

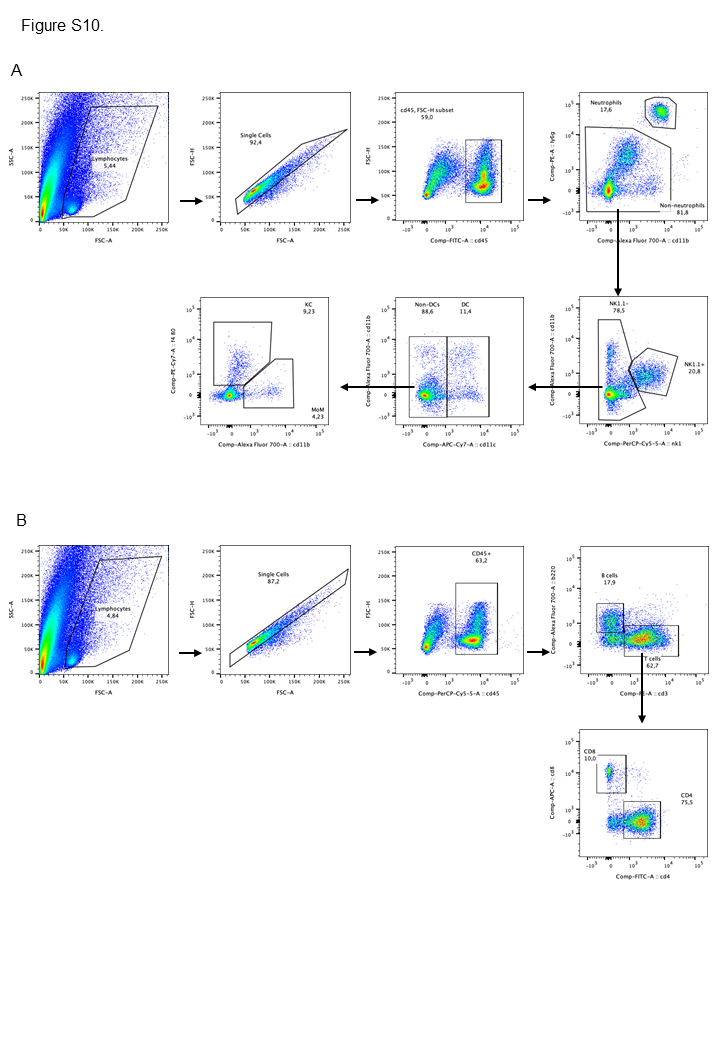

Supplement: Supplementary file 10 — Figure S10: Flow cytometry gating strategies. (A) Representative images of the gating strategy applied to identify hepatic neutrophils, natural killer cells, dendritic cells, monocytes, and Kupffer cells by flow cytometry. (B) Representative images of the gating strategy applied to identify hepatic B cells, T cells, CD4 T helper cells and CD8 cytotoxic T cells by flow cytometry. [file LIV-45-0-s008.png]
