# Supplementary material for: IgM Antibodies Targeting Malondialdehyde Promote Complement‐Mediated Liver Injury in Alcohol‐Related Liver Disease
Source: Liver Int. 2025 Sep 17;45(10):e70356. doi: 10.1111/liv.70356 (PMC12442528; doi:10.1111/liv.70356)
Supplement: Supplementary file 11 — Table S1: Correlation analyses of serum markers in human ALD. [file LIV-45-0-s003.docx]

Table S1: Correlation analyses of serum markers in human ALD.

| **All stages** |  | **MDA-IgM** | **MAA-IgM** | **MDA-IgG** | **MAA-IgG** |
| --- | --- | --- | --- | --- | --- |
| **AST (U/L)** | Spearman r= | 0,2069 | 0,1466 | 0,1628 | 0,2056 |
|  | p-value= | 0,0082 | 0,0627 | 0,0384 | 0,0087 |
| **HVPG (mmHg)** | Spearman r= | 0,2545 | 0,2465 | 0,2239 | 0,235 |
|  | p-value= | 0,001 | 0,0014 | 0,0038 | 0,0024 |
| **CRP (mg/dl)** | Spearman r= | 0,004647 | 0,09079 | 0,08914 | 0,1898 |
|  | p-value= | 0,9529 | 0,2476 | 0,2563 | 0,0149 |
| **IL-6 (pg/ml)** | Spearman r= | -0,005528 | 0,1156 | 0,002479 | 0,1461 |
|  | p-value= | 0,9442 | 0,1415 | 0,9749 | 0,0628 |
| **C3c (mg/dl)** | Spearman r= | -0,1424 | -0,1784 | 0,04408 | -0,0785 |
|  | p-value= | 0,0698 | 0,037 | 0,609 | 0,3619 |
| **C4 (mg/dl)** | Spearman r= | -0,373 | -0,3872 | -0,02923 | -0,06646 |
|  | p-value= | <0,0001 | <0,0001 | 0,7346 | 0,4404 |
| **Compensated** |  | **MDA-IgM** | **MAA-IgM** | **MDA-IgG** | **MAA-IgG** |
| **AST (U/L)** | Spearman r= | 0,03807 | -0,1118 | 0,2188 | 0,3893 |
|  | p-value= | 0,8307 | 0,5292 | 0,2138 | 0,0229 |
| **HVPG (mmHg)** | Spearman r= | 0,03807 | 0,1008 | 0,2188 | 0,3981 |
|  | p-value= | 0,8307 | 0,5644 | 0,2138 | 0,0179 |
| **C3c (mg/dl)** | Spearman r= | -0,1404 | -0,01155 | -0,2335 | -0,3171 |
|  | p-value= | 0,4434 | 0,95 | 0,1983 | 0,077 |
| **C4 (mg/dl)** | Spearman r= | -0,3232 | -0,2758 | -0,2154 | -0,3131 |
|  | p-value= | 0,0711 | 0,1265 | 0,2364 | 0,081 |
| **Decompensated** |  | **MDA-IgM** | **MAA-IgM** | **MDA-IgG** | **MAA-IgG** |
| **AST (U/L)** | Spearman r= | 0,2570 | 0,2276 | 0,1422 | 0,1593 |
|  | p-value= | 0,0034 | 0,0098 | 0,1093 | 0,0724 |
| **HVPG (mmHg)** | Spearman r= | 0,2849 | 0,2981 | 0,2332 | 0,2175 |
|  | p-value= | 0,001 | 0,0006 | 0,0076 | 0,0129 |
| **C3c (mg/dl)** | Spearman r= | -0,1628 | -0,2283 | 0,08798 | -0,02647 |
|  | p-value= | 0,0971 | 0,0192 | 0,3722 | 0,7886 |
| **C4 (mg/dl)** | Spearman r= | -0,3962 | -0,4225 | 0,02239 | 0,01502 |
|  | p-value= | <0,0001 | <0,0001 | 0,8206 | 0,8791 |
