# Supplementary material for: IgM Antibodies Targeting Malondialdehyde Promote Complement‐Mediated Liver Injury in Alcohol‐Related Liver Disease
Source: Liver Int. 2025 Sep 17;45(10):e70356. doi: 10.1111/liv.70356 (PMC12442528; doi:10.1111/liv.70356)
Supplement: Supplementary file 12 — Table S2: Correlation analyses of serum markers in human AUD. [file LIV-45-0-s010.docx]

Table S2: Correlation analyses of serum markers in human AUD.

| **All stages** |  | **MAA-IgM** | **MAA-IgG** |
| --- | --- | --- | --- |
| **AST (U/L)** | Spearman r= | 0,3455 | 0,3005 |
|  | p-value= | 0,0312 | 0,0631 |
